# Binding of small molecules at the P-stalk site of ricin A subunit trigger conformational changes that extend into the active site

**DOI:** 10.1016/j.jbc.2025.108310

**Published:** 2025-02-14

**Authors:** John E. McLaughlin, Michael J. Rudolph, Arkajyoti Dutta, Xiao-Ping Li, Anastasiia M. Tsymbal, Yang Chen, Shibani Bhattacharya, Benjamin Algava, Michael Goger, Jacques Y. Roberge, Nilgun E. Tumer

**Affiliations:** 1 Department of Plant Biology, Rutgers, The State University of New Jersey, New Brunswick, New Jersey, USA; 2 New York Structural Biology Center, New York, New York, USA; 3 Molecular Design and Synthesis Core, Rutgers University Biomolecular Innovations Cores, Office for Research, Rutgers University, Piscataway, New Jersey, USA

**Keywords:** ricin inhibitors, Shiga toxin inhibitors, structure-based design, conformational changes by NMR, ribosome binding

## Abstract

Ricin is a category B agent for bioterrorism, and Shiga toxins are the primary virulence factors of Shiga toxin (Stx) producing *Escherichia coli*. Ricin and Stxs bind the ribosomal P-stalk proteins to depurinate the sarcin/ricin loop on the eukaryotic ribosome and inhibit translation. Both toxins are prime targets for therapeutic intervention because no effective therapy exists for ricin intoxication or Shiga toxin producing *Escherichia coli* infection. Binding of ricin toxin A subunit (RTA) to the ribosomal P-stalk stimulates depurination of the sarcin/ricin loop by an unknown mechanism. We previously identified compounds that bind the P-stalk pocket of RTA and inhibit catalytic activity. Here we characterize a second-generation lead compound, which binds the P-stalk pocket of RTA with over 30-fold improved affinity relative to the original compound and inhibits the cytotoxicity of ricin holotoxin in Vero cells with no apparent cellular toxicity by itself. This compound also shows protection against Stx2A1. X-ray crystal structure of RTA-inhibitor complexes suggests that the orientation of the carboxylic acid influences the inhibitor contacts at the P-stalk site of RTA and contributes to inhibitor potency. The structural changes triggered at the P-stalk site of RTA were validated by solution NMR-based chemical shift perturbation analysis. A key finding by NMR is that binding-induced conformational changes extend beyond the P-stalk site to residues in the active site cleft of RTA. Collectively, these results provide valuable new insight into the conformational flexibility in the C-terminal domain of RTA and its potential role in mediating the remarkable catalytic activity of ricin.

Ricin is one of the most toxic and lethal substances known and is classified as a category-B agent for bioterrorism because of its global availability, stability, and high toxicity (1). Shiga toxins (Stxs) produced by *Escherichia coli* and *Shigella dysenteriae* have the same mechanism of action as ricin (2, 3). Shiga toxins produced by *Escherichia coli* cause gastrointestinal diseases such as hemorrhagic colitis and hemolytic uremic syndrome, which is the most common cause of acute renal failure and brain damage (4, 5, 6). Stxs have two immunologically distinct types, Stx1a and Stx2a. Stx2a is more often associated with progression to hemolytic uremic syndrome (7, 8). Despite years of research, no antidote or vaccine is available against ricin or Stxs. Inhibitors targeting the toxins directly are urgently needed due to the increasing foodborne outbreaks and the emergence of new virulent and multidrug-resistant enterohemorrhagic *E. coli* and *Shigella* strains (9).

Ricin contains an active A subunit (RTA) and a B subunit (RTB), which promotes retrograde transport into the endoplasmic reticulum (ER) (10). RTA is released from RTB in the ER and is retrotranslocated into the cytoplasm (11). Stx2a is an AB_5_ toxin that binds to Gb3 and is internalized by endocytosis. Stx2A is proteolytically cleaved into a catalytic A1 subunit (Stx2A1), which is released from the A2–B5 complex in the ER and undergoes retrotranslocation into the cytosol (11). RTA and Stx2A1 are ribosome inactivating proteins (RIPs), which remove a highly conserved adenine base (A4324 in rat) from the sarcin/ricin loop (SRL) of the 28S rRNA, leading to the inhibition of translation (2, 3), ribotoxic stress (12), and apoptosis (13).

RIPs have evolved to be near perfect catalysts for mammalian ribosomes (14, 15). RTA binds eukaryotic ribosomes with very high speed (16, 17) and depurinates ribosomes at a rate of 1500 ribosomes per min per RTA (18). We showed that RTA is recruited to the SRL by binding first to a conserved sequence at the C-terminal domain (CTD) of ribosomal P-stalk proteins (19, 20). The interaction of RTA with the P-stalk is of high biological importance because RTA must compete with the elongation factors for binding the P-stalk. The eukaryotic ribosomal P-stalk is a pentameric protein complex formed by uL10 protein (P0) and two P1-P2 heterodimers attached to uL10 (21, 22). The entire structure of the P-stalk has not been solved due to its flexibility. A unique feature of the eukaryotic P-stalk is that all five P-proteins have unstructured C-termini with identical 11 amino acids (P11: SDDDMGFGLFD) (22). The P-stalk CTD recruits the translational GTPases such as the elongation factors to the ribosome and stimulates their GTPase activity (21, 22, 23). The X-ray crystal structure analysis showed that RTA binds the last six amino acids of P11 in a hydrophobic pocket at its CTD (24, 25). The P-stalk binding surface of RTA is on the opposite side of the active site cleft (26). RTA cannot depurinate a stem-loop RNA mimicking the SRL at pH 7.0 (14) but depurinates ribosomes at pH 7.0 because binding to the ribosomal P-stalk is necessary for depurination of the SRL (19, 20). P-stalk binding stimulates the depurination of the SRL by RTA on the ribosome ∼200-fold at the physiological pH (26, 27). The enhancement in the depurination activity observed in the presence of the pentameric P-stalk proteins is not solely attributed to a concentration effect. Therefore, an unresolved question in the field is the nature of the communication between the P-stalk binding site and the active site of RTA, which are separated by ∼20 Å.

The active site of RTA has been a primary target for antidote development. The majority of RTA inhibitors reported to date are substrate analogs that target the active site with low affinity and solubility (28, 29, 30). Most of these compounds were pterin-based molecules that fit into the active site (30, 31, 32, 33). These inhibitors did not show activity in cell and animal models of toxicity (28, 29, 30). Transition state analogs inhibited depurination of small stem-loop RNA substrates at low pH but did not protect eukaryotic ribosomes at physiological pH (34). A retrograde transport blocker, Retro-2 had activity against ricin and Stx1a in cell-based assays and showed partial protection of mice against ricin by blocking retrograde trafficking (35, 36).

Targeting toxin–ribosome interactions by small molecules is a significant challenge and has not been explored as a strategy for the inhibition of ricin or Stx2a. We used fragment-based lead discovery with surface plasmon resonance and identified CC10501, which binds the P-stalk binding site of RTA and reduces catalytic activity (37). We improved CC10501 using structure-based design and identified RU-NT-206, which bound the P-stalk pocket of RTA with submicromolar affinity and protected mammalian cells from ribosome depurination by ricin holotoxin (38). Here we evaluated the second-generation lead compounds that bind at the P-stalk binding site of RTA and identified RU-NT-192, which had 30-fold improved affinity relative to CC10501 and inhibited the cytotoxicity of ricin holotoxin in mammalian cells with no apparent cellular toxicity by itself for the first time. RU-NT-192 also showed protection against Stx2A1. Analysis of the binding mode of RTA-RU-NT-192 and RTA-RU-NT-206 complexes by X-ray crystallography revealed the critical interactions that may contribute to inhibitor potency. To evaluate the binding mechanism and conformational influence of RU-NT-192 and RU-NT-206, we used solution NMR and applied chemical shift perturbation (CSP) assay to RTA bound to each inhibitor. Our results provide the first evidence that binding of RU-NT-192 and RU-NT-206 induce conformational changes at the P-stalk site of RTA that allosterically affect the active site.

## Results

### Structure-activity relationship of the inhibitors

The crystal structure revealed that CC10501 binds RTA (Table 1, PDB: 6URX) in a similar way as P11 (PDB: 5GU4) forming comparable interactions (37). We used structure-based design to optimize the CC10501 chemical scaffold and discovered RU-NT-93 (Table 1, PDB: 7MLP) and RU-NT-102 (Table 1, PDB: 7MLT) with improved affinity and potency (39). RU-NT-93 and RU-NT-102 similarly bound in the P-stalk pocket as CC10501 and protected Vero cells against ricin holotoxin over 10-fold more effectively than CC10501 (39). To make RU-NT-93 fit better into the hydrophobic pocket, we synthesized structural analogs of RU-NT-93 by introducing different functional groups and measured the binding affinity and the 50% inhibitory concentration (IC_50_) of the analogs using a fluorescence anisotropy (FA)-based competitive binding assay (40). The FA assay measures the decrease in FA caused by inhibitors that displace the fluorescent P11 peptide, which binds at the P-stalk site of RTA (40). The IC_50_ value is the inhibitor concentration required to replace 50% of the fluorescent P11 probe from RTA. The inhibition constant (*K*_i_) is the inhibitor concentration that will bind to half the binding sites on RTA at equilibrium and is calculated from the IC_50_ values (40). Among the ∼50 RU-NT-93 analogs analyzed, those with the highest affinity are shown in Table 1. The *K*_i_ values of the previously reported RU-NT-93 (39), RU-NT-102 (39), and RU-NT-206 (38) are also included for comparison using a more sensitive BioTek Synergy H1 plate reader (Fig. S1). RU-NT-192 had a 32-fold improved *K*_i_ value relative to CC10501 (Table 1), while RU-NT-206 showed a 53-fold improved *K*_i_ relative to CC10501 (Table 1) (38). RU-NT-192 had a 2.6-fold lower *K*_i_ value than the 5-fold larger P11 peptide (Fig. S1). The ligand efficiency values, which represent the binding energy divided by the number of nonhydrogen atoms of each compound were ∼ 0.4, indicating a good starting point for hit development since most drugs have ligand efficiency > 0.3 (41).

**Table 1:** Chemical structures, affinity, and potency of the CC10501 analogs

RU-NT	Structure	MW (Da)	LE^a^	*K*_i_^b^ (μM)	IC_50_^c^ (μM)	EC_50_^d^ (μM)	Viability^e^ (pM)
CC10501		204	0.44	32	427 n = 1.7^f^	UD	UD
93		232	0.47	3	40 n = 1	UD	8
102		232	0.49	2	38 n = 1.3	120 n = 1	7
202		250	0.44	4	32 n = 1	169 n = 1	20
165		246	0.46	2	45 n = 1.5	98 n = 1.5	50
192		260	0.46	1	18.5 n = 1.2	38	200
206		258	0.47	0.6	18 n = 1.6	29 n = 1	UD

UD, unable to determine.

a The ligand efficiency (LE) values represent the binding energy divided by the number of nonhydrogen atoms of each compound.

b The *K*_i_ values were measured by fluorescence anisotropy (FA) using a BioTek Synergy H1 plate reader and calculated from the IC_50_ values, which represent displacement of the fluorescent P11 probe from RTA.

c The IC_50_ value represents inhibition of *in vitro* depurination of rat liver ribosomes by RTA as determined by qRT-PCR.

d The depurination EC_50_ value is the half-maximal concentration required to inhibit depurination by ricin holotoxin in Vero cells determined by qRT-PCR.

e The viability EC_50_ values represent the amount of ricin holotoxin that reduces the viability of Vero cells by 50% in the presence of 250 μM of each compound at 48 h.

f The “n” values represent the Hill coefficient derived by fitting the data using the Hill equation.

**Figure 1:**
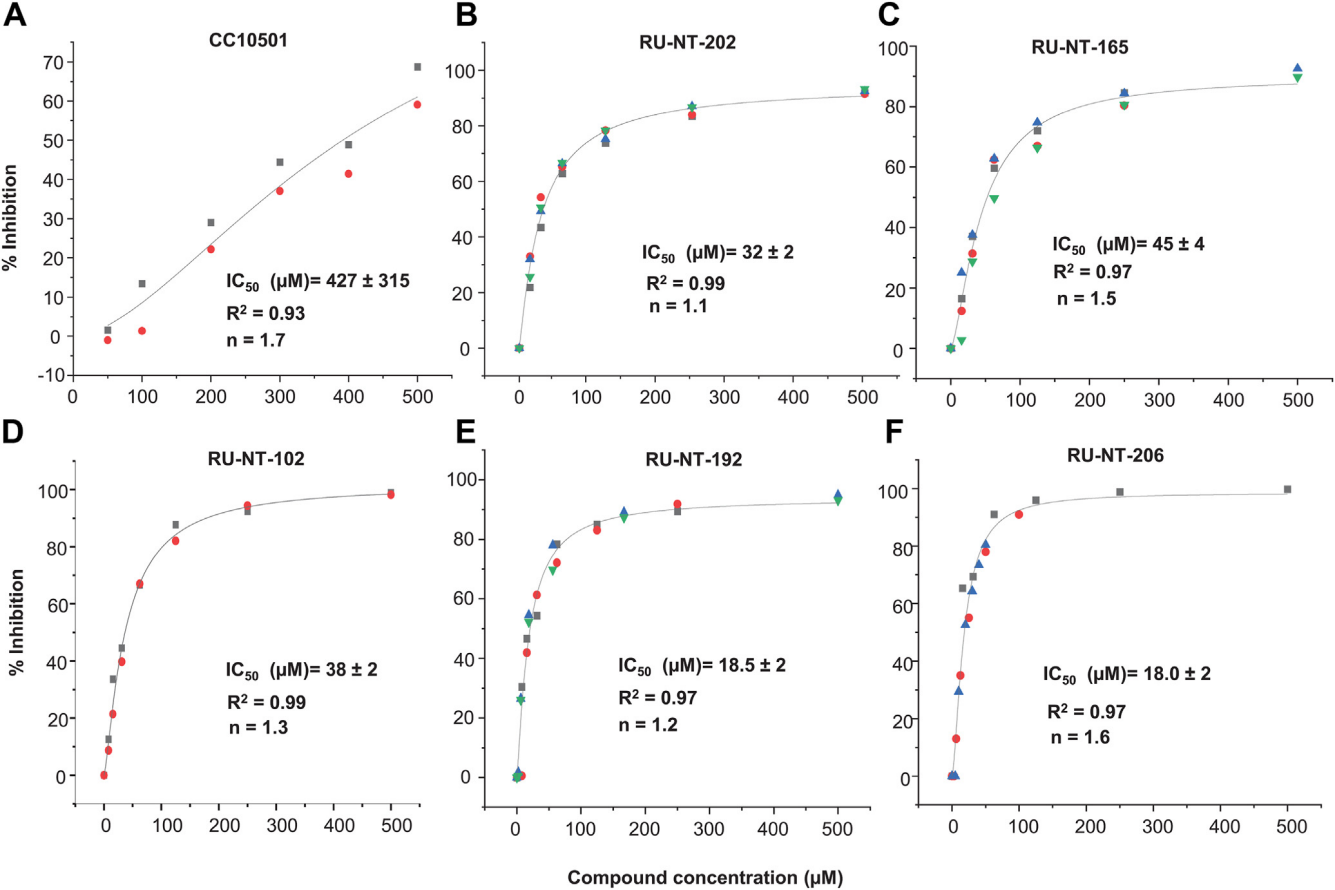
**Inhibition ofRTA-mediateddepurination of rat liver ribosomes.** CC10501 (*A*), RU-NT-202 (*B*), RU-NT-165 (*C*), RU-NT-102 (*D*), RU-NT-192 (*E*), and RU-NT-206 (*F*) at different concentrations were mixed with 200 pM RTA first and rat liver ribosomes were added to start the reaction. The reaction was incubated at room temperature for 5 min. RNA extraction buffer was added to stop the reaction. RNA was extracted and the level of inhibition was determined by qRT-PCR. Measurements were repeated 2 to 4 times as indicated by the different symbols. The data for the percentage of inhibition at different compound concentrations were fitted with the Hill equation using OriginPro 2023 to calculate the IC_50_. The “n” values represent the Hill coefficient.

The *in vitro* activities of the analogs were determined by their ability to inhibit depurination of rat liver ribosomes by RTA *in vitro* using quantitative real-time PCR (qRT-PCR) (42, 43) and the data were fit using the Hill equation (44) (Fig. 1). The IC_50_ value measured by qRT-PCR is the inhibitor concentration required to inhibit RTA-mediated depurination of rat liver ribosomes by 50% *in vitro*. RU-NT-192 had a 23-fold improved IC_50_ by qRT-PCR relative to CC10501 similar to RU-NT-206, which had a 24-fold improved IC_50_ relative to CC10501 (Table 1). The Hill coefficient (n) of RU-NT-192 was close to one, indicating 1:1 interaction with RTA (Fig. 1 and Table 1).

### Protection in the cell-based assay

**Figure 2:**
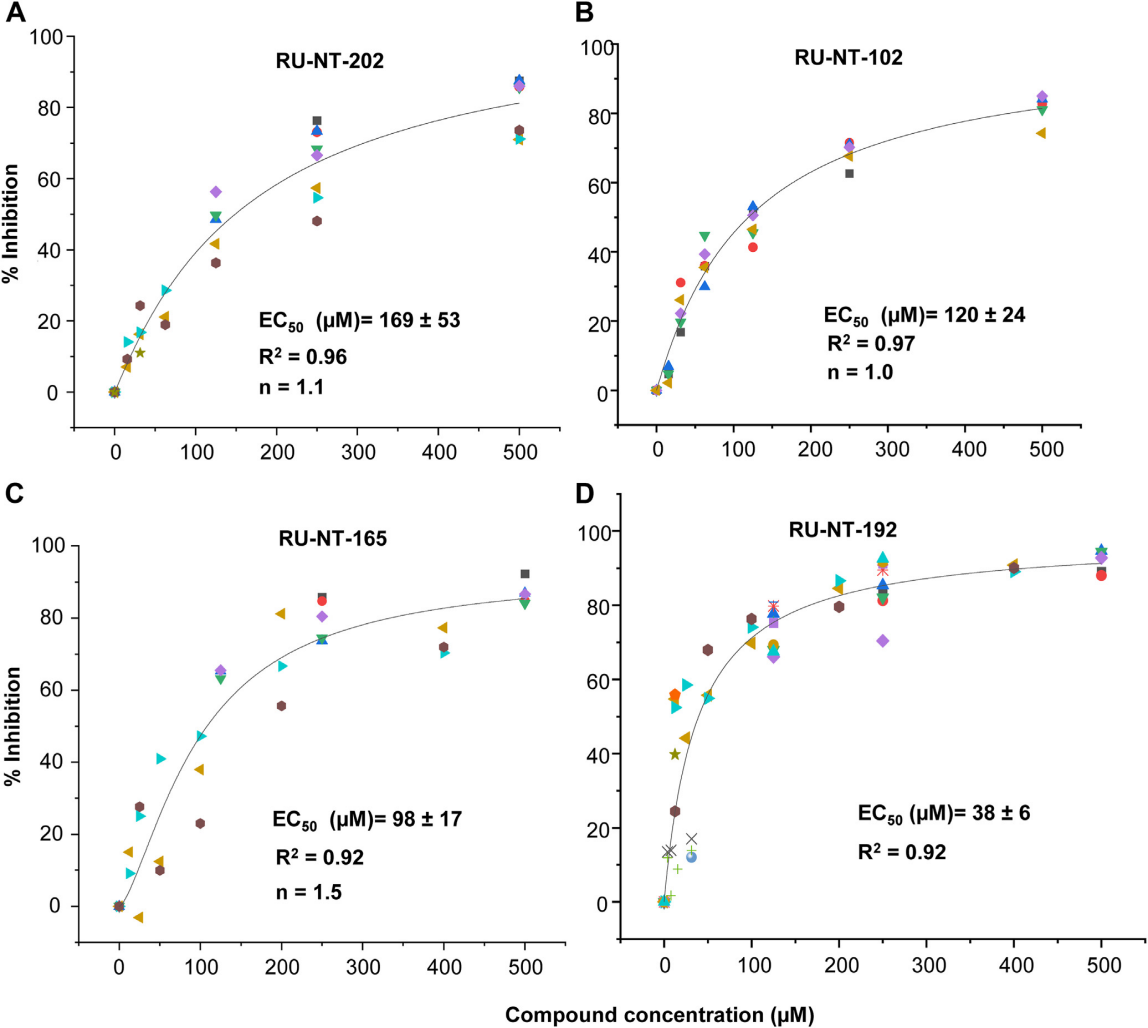
**Inhibition of ribosome depurination by ricin holotoxin in Vero cells.** Vero cells were plated at 1.5 × 10^5^ /ml and grown for 24 h. Cells were treated with RU-NT-202 (*A*), RU-NT-102 (*B*), RU-NT-165 (*C*), and RU-NT-192 (*D)* and 200 pM ricin as described in the Experimental procedures. The percentage of depurination was measured by qRT-PCR compared to DMSO-treated cells at 2 h. The half maximal effective concentration (EC_50_) for inhibition of depurination by ricin holotoxin in Vero cells was determined by fitting the data either with the Hill equation (*A*–*C*) or the Michaelis–Menten equation (*D*) using OriginPro 2023. Data are from 3 to 16 biological replicates shown in different colors. The “n” values represent the Hill coefficient.

We examined the effect of the improved compounds on the depurination activity and cytotoxicity of ricin holotoxin in Vero cells. In the presence of 200 pM ricin holotoxin, ribosome depurination increased linearly for 4 h. Two hours after the simultaneous addition of ricin holotoxin and the compound, cellular RNA was isolated and used for qRT-PCR to determine the level of depurination. We were able to determine the half-maximal effective concentration (EC_50_) of depurination inhibition by ricin holotoxin for RU-NT-102, RU-NT-202, RU-NT-165, and RU-NT-192 by incubation with a titration series of each compound. The compounds alone did not cause any cytotoxicity to Vero cells up to 500 μM. The EC_50_ values were determined by fitting the data using the Hill equation (Fig. 2). The EC_50_ of RU-NT-206 was 29 ± 2 μM and n = 1 using the Hill equation as previously reported using the Michaelis–Menten model (38). RU-NT-192 protected Vero cells from depurination by ricin holotoxin with an EC_50_ of 50 ± 18 μM and n = 1 by fitting the data with the Hill equation. The RU-NT-192 protection data fit the Michaelis–Menten equation better with an EC_50_ value of 38 ± 6 μM (Fig. 2).

To determine if there is a correlation between the *K*_i_ values and protection in the cell-based assay, the *K*_i_ values were plotted against the Vero cell EC_50_ values in Fig. S2. The plot indicates that the *K*_i_ values of the inhibitors in Table 1 are directly proportional to the EC_50_ values for inhibition of depurination in Vero cells (Pearson's r = 0.96), indicating that the *K*_i_ is a good indicator of cellular potency and can be used to distinguish between the inhibitors and to rank them.

**Figure 3:**
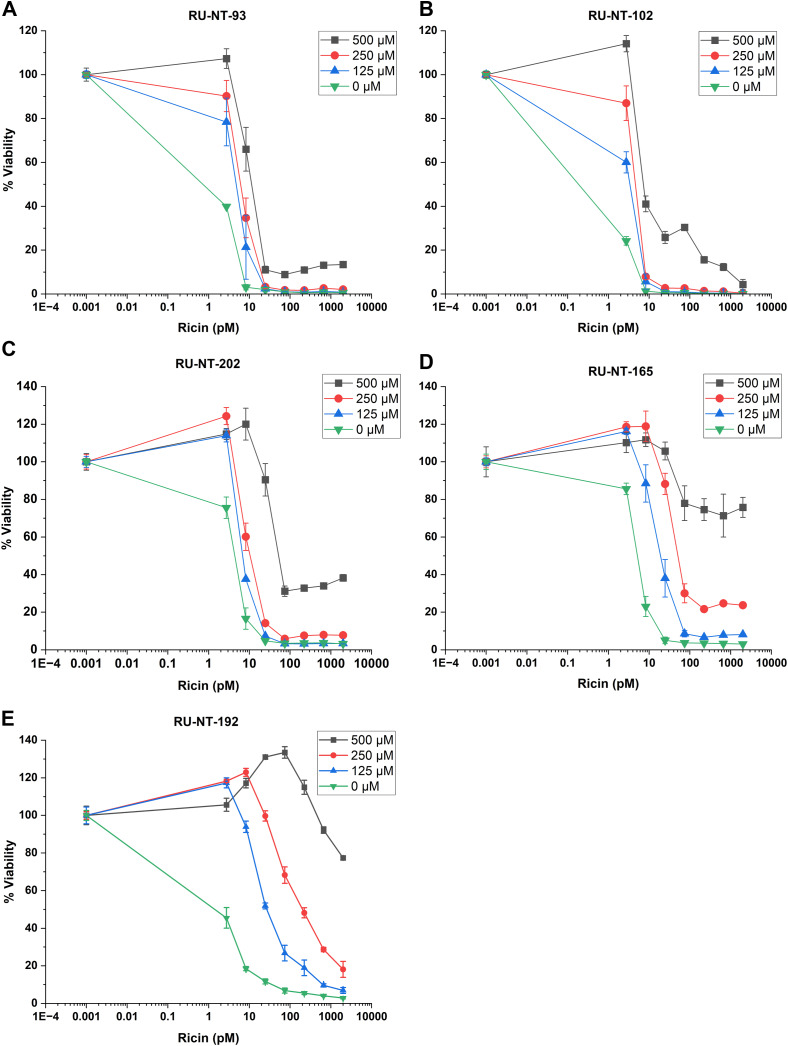
**Protection against the cytotoxicity of ricin holotoxin.** Protection by RU-NT-93 (*A*), RU-NT-102 (*B*), RU-NT-202 (C), RU-NT-165 (*D*), and RU-NT-192 (*E*) in Vero cells was determined by measuring Vero cell viability at 48 h using the CellTiter-Glo Luminescent Cell Viability Assay (Promega), which measures the ATP content released from lysed cells. The data represent three biological replicates.

To determine if reduced ribosome depurination by ricin holotoxin in Vero cells would translate into protection of cell growth and viability, we examined the viability of Vero cells exposed to ricin holotoxin using the CellTiter-Glo Luminescent Cell Viability Assay (Promega), which measures the ATP content released from lysed cells. Vero cells grown for 24 h were exposed to seven different concentrations of ricin up to 2000 pM and different concentrations of RU-NT-93, RU-NT-102, RU-NT-202, RU-NT-165, and RU-NT-192 and a negative control in the absence of the compound. After 48 h of growth, Cell-Titer-Glo reagent was added, and the luminescence was read using an Agilent BioTek Synergy H1 plate reader. The compounds by themselves did not cause any reduction in viability in Vero cells at the concentrations tested. The amount of ricin that reduced the viability of Vero cells by 50% at 48 h in the absence of any compound was defined as the EC_50_. As shown in Figure 3, ∼1 pM ricin reduced the viability of Vero cells by ∼50% in the absence of any compound. RU-NT-93 and RU-NT-102 protected cells against the cytotoxicity of ricin with EC_50_ values of 8 and 7 pM at 250 μM, respectively (Fig. 3, *A* and *B* and Table 1). Treatment with RU-NT-202 led to a 2-fold, 5-fold, and 15-fold increase in EC_50_ at 125, 250, and 500 μM respectively, relative to ricin alone with no compound (Fig. 3*C*). RU-NT-202 had an EC_50_ value of 20 pM at 250 μM (Table 1). RU-NT-165 improved the EC_50_ by 5-, 12.5-, and >500-fold at 125, 250, and 500 μM, respectively, relative to ricin alone with no compound (Fig. 3*D*). RU-NT-165 had an EC_50_ value of 50 pM at 250 μM (Table 1). RU-NT-192 improved the EC_50_ by 10-fold at 125 μM and by 200-fold at 250 μM and had an EC_50_ value of 200 pM at 250 μM (Table 1). The viability remained at about 80% at 2000 pM ricin, indicating >1000-fold improvement when RU-NT-192 was used at 500 μM (Fig. 3*E*). Treatment with RU-NT-206 at all concentrations interfered with the viability assay and was not included in this dataset. These results demonstrated that the improved compounds protected Vero cells from the cytotoxicity of ricin holotoxin in agreement with their potencies for the inhibition of ribosome depurination in the cell-based assay (Fig. 2). RU-NT-192 showed the greatest protection against the cytotoxicity of ricin holotoxin with no apparent cellular toxicity.

### X-ray crystal structure of the RTA-inhibitor complexes

**Figure 4:**
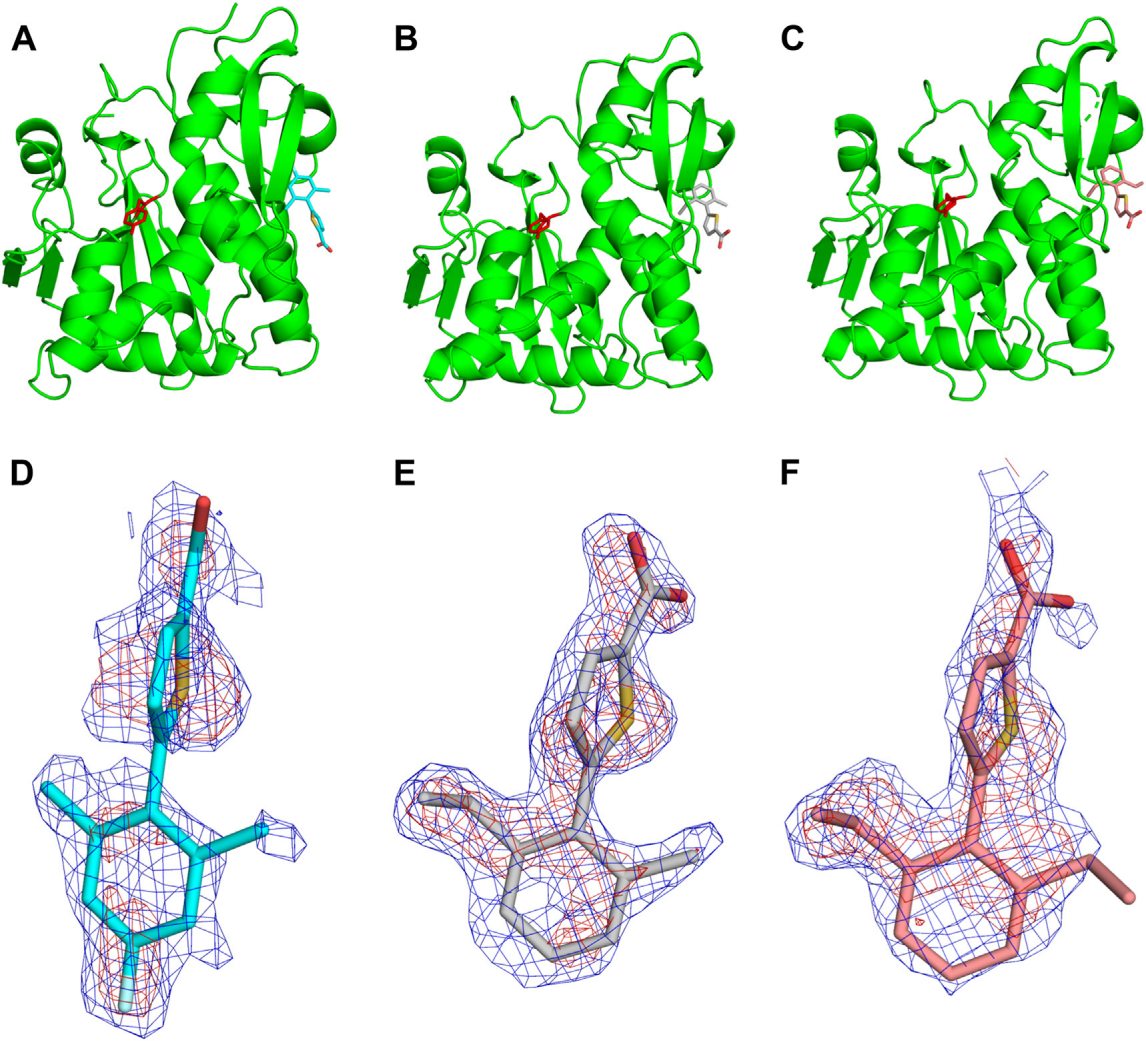
**Structures ofRTA-inhibitorcomplexes.** Structure of RTA (*green*) depicted as a ribbon diagram in complex with inhibitors (*A*) RU-NT-202 (*cyan*), (*B*) RU-NT-165 (*gray*), and (*C*) RU-NT-192 (*salmon red*). RTA active site residue Tyr80 is drawn as sticks and colored *red*. 2Fo-Fc (*blue* mesh) and Fo-Fc (*red mesh*) electron density omit maps of (*D*) RU-NT-202, (*E*) RU-NT-165, and (*F*) RU-NT-192. The original 2Fo-Fc and Fo-Fc electron density omit maps were contoured at 1.0 σ and 3.0 σ levels, respectively. The omit maps were calculated before each inhibitor was built into the density maps. Each inhibitor is drawn as sticks with all carbon atoms in RU-NT-202 colored *cyan*, all carbon atoms in RU-NT-165 colored *gray*, and all carbon atoms in RU-NT-192 colored *salmon red*. All oxygen atoms are colored *red* and sulfur atoms *yellow*.

RTA was cocrystallized with RU-NT-202, RU-NT-165, and RU-NT-192 and each RTA-inhibitor complex structure was solved by molecular replacement using PHASER (Fig. 4, *A*–*C*). The RTA-RU-NT-202, RTA-RU-NT-165, and RTA-RU-NT-192 structures were all determined at 1.8 Å. The RTA-RU-NT-165 and RTA-RU-NT-192 structures were determined in the monoclinic P2_1_ space group with the RTA-RU-NT-202 complex structures solved in the orthorhombic P2_1_2_1_2_1_ space group. The electron density for each inhibitor was well-defined in the omit electron density maps (Fig. 4, *D*–*F*) and in the fully-refined electron density maps for each structure (Fig. S3, *A*–*C*).

**Figure 5:**
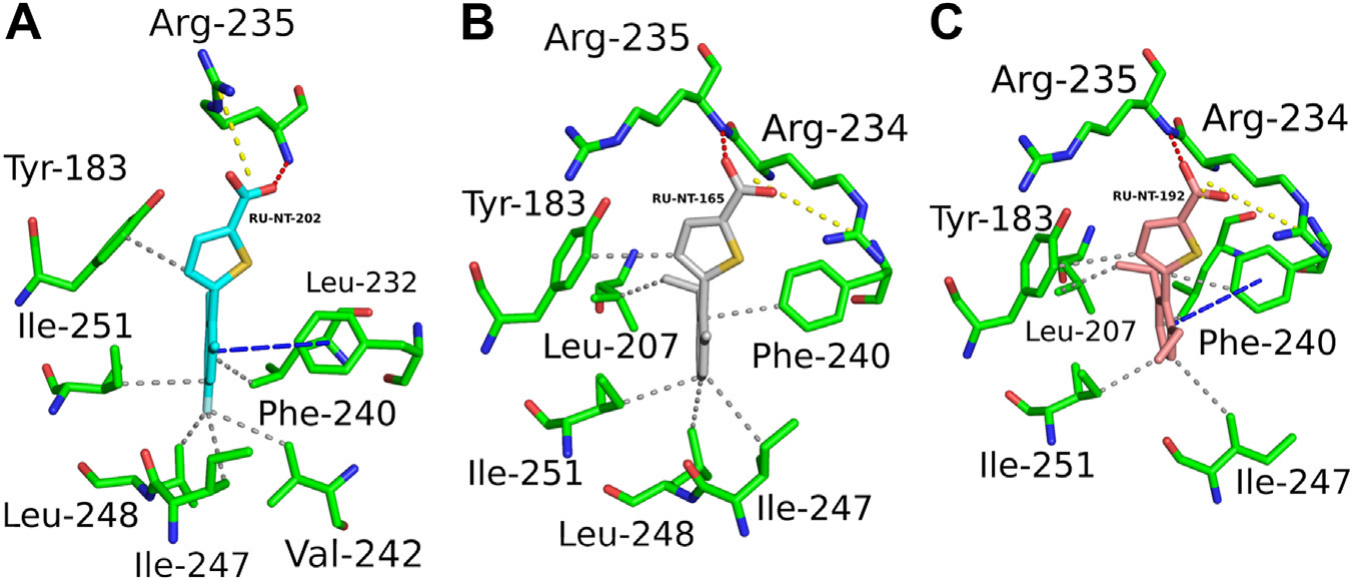
**Key inhibitor interactions with RTA.** Zoom-in of the noncovalent interactions of RTA (*green*) in complex with (*A*) RU-NT-202 (*cyan*), (*B*) RU-NT-165 (*gray*), and (*C*) RU-NT-192 (*salmon red*) all drawn as *sticks*. All nitrogen atoms were colored *blue*, all oxygen atoms were colored *red*, and all sulfur atoms were colored *yellow*. The fluorine atom on RU-NT-202 is colored *pale cyan*. The salt bridges and hydrogen bond are represented as *yellow* and *red dashes*, respectively, with the similar nonpolar contacts between RTA and each inhibitor represented as *gray dashes*. The π–π interactions are drawn as *blue dashes*.

The binding positions of RU-NT-202, RU-NT-165, and RU-NT-192 within the RTA P-stalk pocket are closely related (Fig. S4). After superimposing these structures and the structure of RTA with RU-NT-206 (38), the RMSD values range from 0.23 to 0.46 Å, demonstrating no significant conformational changes in the RTA backbone upon binding of these inhibitors (Fig. S5). Both RU-NT-165 and RU-NT-192 exhibited more comparable binding patterns, where the carboxylate group within the thiophene ring of each inhibitor formed a salt bridge with the Arg234 side chain and a hydrogen bond with the main chain amide nitrogen of Arg235. In contrast, the thiophene carboxylate in RU-NT-202 only interacted with Arg235, forming a salt bridge with the side chain and a hydrogen bond with the main chain of Arg235. (Fig. 5, *A*–*C*). The thiophene ring in all three inhibitors established a similar hydrophobic interaction with Tyr183. The benzene rings in all three compounds formed a network of hydrophobic interactions with Tyr183 and Phe240 in RTA, with RU-NT-192 and RU-NT-202 additionally engaging in π-π stacking interactions with Phe240. Each benzene ring hydrophobically contacted Ile247, Leu248, and Ile251 in RTA (Fig. 5, *A*–*C*). Moreover, the ethyl groups at the 2-position within the benzene rings from RU-NT-165 and RU-NT-192 formed distinct hydrophobic interactions with Leu207 (Fig. 5, *B* and *C*). The fluorine atom of the fluorobenzene ring in RU-NT-202 uniquely interacted with Val242 in RTA, while making closer contacts with Ile247 and Leu248 (Fig. 5*A*). Additionally, in the RTA-RU-NT-165 and RTA-RU-NT-192 structures, the carboxylate group from one of the inhibitors formed a spurious salt bridge with Arg234 from the second RTA copy within the asymmetric unit.

**Figure 6:**
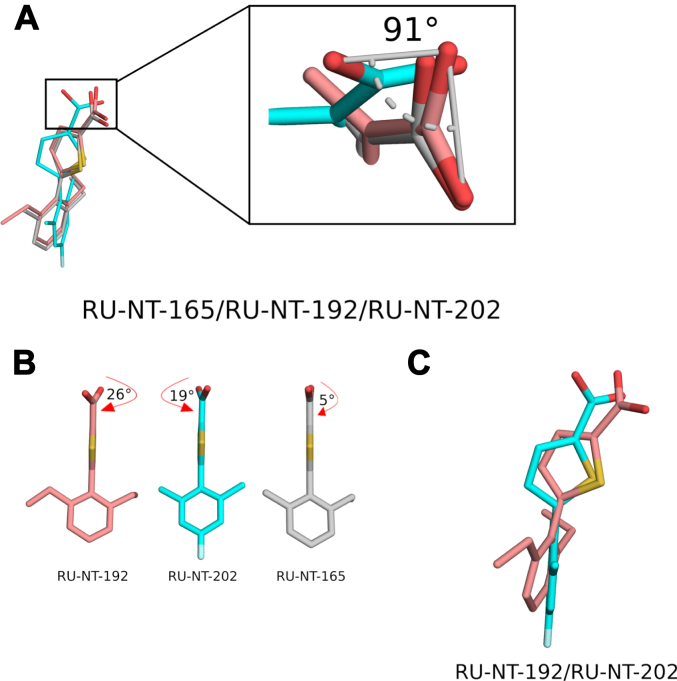
**Carboxylate configuration influences inhibitor effectiveness.***A*, a zoomed-in view of the relative rotation of the carboxylate group in RU-NT-202 of 91° relative to the carboxylate group in RU-NT-192 and RU-NT-165, which distanced the carboxylate group of RU-NT-202 away from Arg234 in RTA precluding noncovalent interaction with this RTA residue. *B*, RU-NT-192 (*salmon red*), RU-NT-202 (*cyan*), and RU-NT-165 (*gray*) *drawn as sticks* depicting the relative orientation of the carboxylate relative to the thiophene ring in each inhibitor where the larger angle of 26° in RU-NT-192 and 19° in RU-NT-202 slightly increases the electron density around the carboxylate compared to RU-NT-165. The *red arrow* highlights the rotation of the carboxylate group relative to the thiophene ring in each inhibitor. *C*, RU-NT-192 (*salmon red sticks*) superposed onto RU-NT-202 (*cyan sticks*) revealing the different positions of the benzene ring in each inhibitor.

Although all three inhibitors bound similarly to the P-stalk pocket, RU-NT-202 adopted a notably different orientation, rotating its carboxylate moiety 91° compared to RU-NT-192 and RU-NT-165 (Fig. 6*A*). This shifted the thiophene carboxylate position in RU-NT-202 ∼2 Å away from Arg234, thereby precluding any interaction with this residue and potentially reducing binding affinity for RTA. Furthermore, the dihedral angles between the carboxylate group and the thiophene ring were 26.1° for RU-NT-192 and 19.0° for RU-NT-202, whereas RU-NT-165 featured a nearly coplanar orientation of the thiophene ring to its carboxylate group with a minimal rotation of 5° (Fig. 6*B*). The larger dihedral angle between the carboxylate and thiophene rings in RU-NT-192 and RU-NT-202 leads to decreased conjugation and heightened electron density in the carboxylate group, strengthening ionic interactions of RU-NT-192 with the positively charged arginines, potentially improving its binding affinity. While for RU-NT-202, a more negatively charged carboxylate enhances the interaction exclusively with Arg234, indicating that RU-NT-192 benefits more significantly from the increased negative charge on its carboxylate. In RU-NT-165, the carboxylate being nearly coplanar with the thiophene spreads the charge across both the thiophene and the carboxylate, potentially reducing the ionic interactions with both Arg234 and Arg235 compared to RU-NT-192. The fluorine atom in the fluorobenzene ring of RU-NT-202 interacted specifically with several nonpolar residues lining the P-stalk pocket (Fig. 5*A*), causing a shift in the position of the benzene ring in RU-NT-202 compared to RU-NT-192 (Fig. 6*C*), aligning the thiophene ring and carboxylate group of RU-NT-202 away from Arg234.

### NMR analysis of the RTA-inhibitor complexes

**Figure 7:**
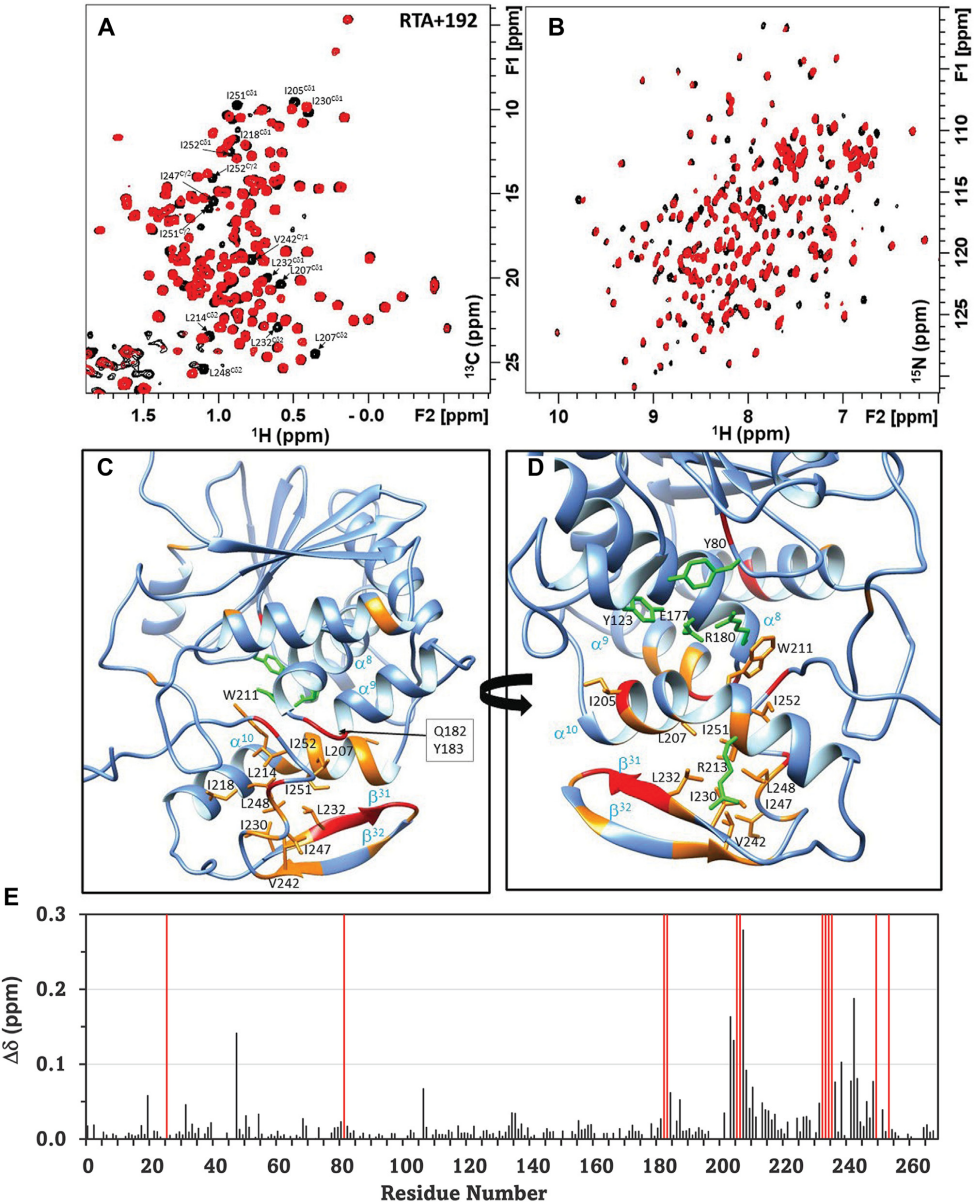
**Analysis of RU-NT-192 binding site in RTA using chemical shift perturbation data obtained from NMR experiments acquired at 700 MHz spectrometer and 25 °C temperature.***A*, methyl ^1^H-^13^C HMQC and (*B*) amide ^1^H-^15^N HSQC in the free state of RTA (*black contours*) overlaid with the inhibitor-bound complex (*red contours*). *C* and *D*, backbone CSPs mapped on the X-ray structure of RTA (PDB code 1RTC) in two different orientations. Residues with amide CSPs > 0.05 ppm are highlighted in *orange* and those that disappear in the complex due to exchange broadening are painted *red*. Annotated methyl groups with CSP > 0 from *panel A*; substrate binding and catalytic site residues (*green*) are shown in line representation. *E*, residue-specific profile of the weighted average of the amide proton (^1^H) and nitrogen (^15^N) chemical shift differences between the free state and inhibitor complex calculated using the relationship √0.5∗[(Δd_H_^N^)^2^ + (0.14∗Δd_N_)^2^]. The exchange-broadened sites in the complex are indicated by *red lines*.

**Figure 8:**
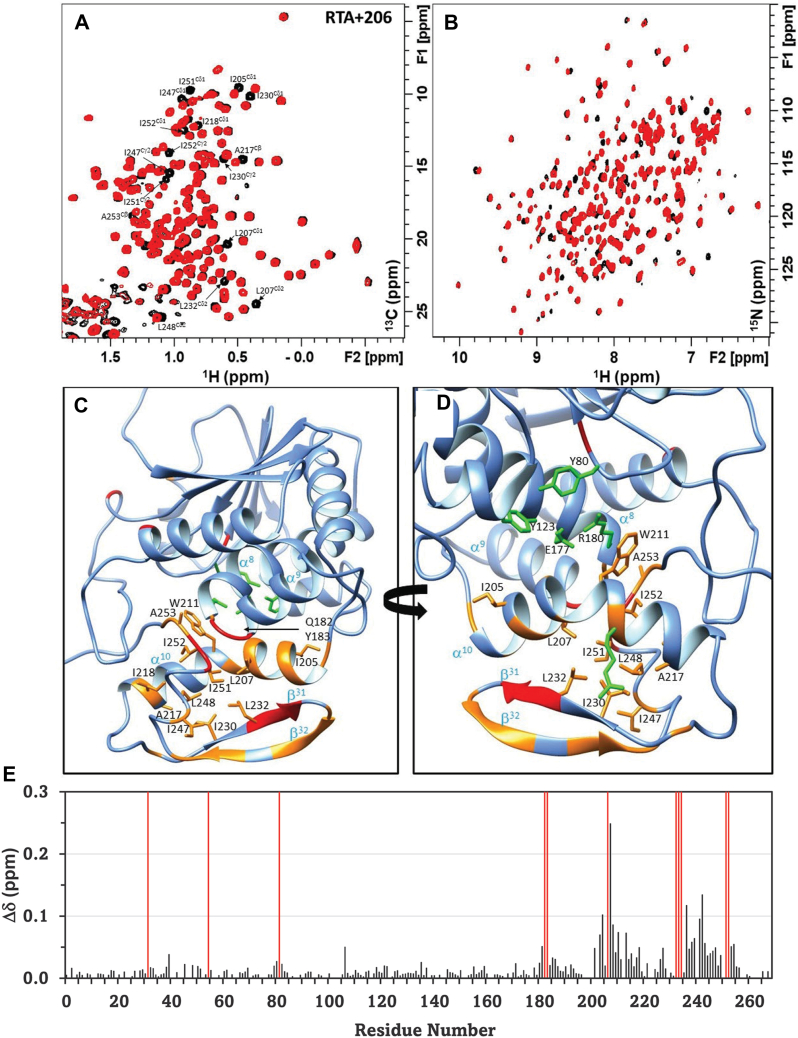
**Analysis of RU-NT-206 binding site in RTA using chemical shift perturbation data obtained from NMR experiments acquired at 700 MHz spectrometer and 25 °C temperature.***A*, methyl ^1^H-^13^C HMQC and (*B*) amide ^1^H-^15^N HSQC in the free state of RTA (*black contours*) overlaid with the inhibitor-bound complex (*red contours*). *C* and *D*, backbone CSPs mapped on the X-ray structure of RTA (PDB code 1RTC) in two different orientations. Residues with amide CSPs > 0.05 ppm are highlighted in *orange* and those that disappear in the complex due to exchange broadening are painted *red*. Annotated methyl groups with CSP > 0 (*panel A*) and the catalytic site residues (*green*) are shown in line representation. *E*, residue-specific profile of the weighted average of the amide proton (^1^H) and nitrogen (^15^N) chemical shift differences between the free state and inhibitor complex calculated using the relationship √0.5∗[(Δd_H_^N^)^2^ + (0.14∗Δd_N_)^2^]. The exchange-broadened sites are shown by *red lines*.

The crystal structure analysis suggested that compounds effectively disrupting the strongest contacts between the P-stalk proteins and key residues within the P-stalk pocket of RTA more potently block the interaction with the ribosome, resulting in more effective inhibition of ricin. The X-ray crystal structure analysis failed to detect any significant conformational changes in the backbone of RTA upon binding of these inhibitors (Fig. S5). CSP is a complementary approach to validate the inhibitor binding sites identified in the X-ray structures and probe concurrent conformational changes (45, 46). To harness this information, we previously published the backbone amide (NH) and methyl group (CH_3_) chemical shift assignments of the 267 residue RTA in the free state and in complex with the P11 peptide (47). Here we applied CSP analysis to RTA bound to RU-NT-192 and RU-NT-206 to characterize the binding-induced structural changes in the enzyme. The residues that exhibit the largest CSPs in the presence of RU-NT-192 (Fig. 7) and RU-NT-206 (Fig. 8) are clustered in the P-stalk site of RTA. The similarity of the CSP profiles of the two inhibitors with only minor differences reflects a common mode of binding involving hydrophobic side chains (Ile247, Ile251, Tyr183, Phe240) augmented by the formation of salt bridges with the charged side chains (Arg234, Arg235) (Fig. 5). This conclusion is widely supported by the modest CSPs in the methyl spectrum of RTA, which is consistent with a repacking of the hydrophobic core in the CTD (Figs. 7 and 8, *A* and *B*). We see some evidence of conformational exchange (48) along the protein backbone bearing the arginine side chains, which leads to line broadening of residues in the α^8^-α^9^ turn (182–183) and β^31^ (232–234) strand (Figs. 7 and 8, *C*–*E*).

The extensive perturbation of both backbone and side-chain chemical shifts along helix α^10^ (202–220), strands β^31^ (225–233) and β^32^ (239–244) suggests that the CTD undergoes a structural change that extends beyond the key interaction residues. In the N-terminal domain, we see little evidence of structural change in the inhibitor-bound complex. The notable exception includes residues (184, 187, 208, 210, 213, and Trp-211 NE1) located at the interface between the α9 and α10 helices (Figs. 7, and 8, *D* and *E*). These residues are not directly involved in binding the inhibitor but play a vital role in binding the adenine substrate (49, 50, 51, 52, 53).

## Discussion

Ricin and Stxs have been uniquely challenging drug targets. There is a significant need for developing antidotes against ricin and Stxs because there are no vaccines or prophylactic monoclonal antibodies that have received approval. Despite decades of work, there is no inhibitor targeting the active site that neutralizes the cytotoxicity of ricin (28, 29, 30). Small molecule inhibitors targeting the active site, which are based on pterins, guanines, and pyrimidines, inhibited RTA with high micromolar IC_50_ values and had solubility, toxicity, and cell permeability issues (30, 31, 32, 33). None of these inhibitors showed protection against ricin in cell-based assays (28, 29, 30). In our fragment screens against RTA and Stx2A1, we identified compounds that bind the P-stalk site but not the active site of either toxin, suggesting that the active site is a less suitable target (37, 54). We showed that blocking the ribosome-binding site with peptide mimics of the P-stalk CTD or small molecule fragments inhibits the catalytic activity of RTA (37, 55) and Stx2A1 (54, 56). The ability of these compounds to inhibit depurination at the active site, which is separated from the P-stalk site by ∼20 Å demonstrates the importance of the P-stalk binding site as a novel target for designing antidotes.

RU-NT-206 was identified as a lead compound, which bound the P-stalk pocket of RTA with similar affinity as a five-fold larger P-stalk peptide, P11, and protected cells against ricin and Stx2 holotoxins, validating the P-stalk binding site of RTA as a druggable target (38). Here we identified RU-NT-192 as a lead compound, which had 2.6-fold higher affinity than the P11 peptide and inhibited the cytotoxicity of ricin holotoxin in Vero cells with no apparent cellular toxicity. Our results highlight the complementarity of the X-ray and the NMR data and provide evidence for the role of the conformational flexibility of RTA in binding different inhibitors. We show that binding-induced allosteric changes extend beyond the P-stalk binding site into the active site of RTA. These results provide new insight into the P-stalk interactions, which may contribute to the allosteric regulation of the depurination activity of RTA.

The crystal structure of RTA bound to the P11 peptide and P-stalk inhibitors shows that the peptide binds to the P-stalk pocket similarly to the inhibitors (Fig. S6*A*). Notably, the C-terminal Asp11 of P11 interacts with the two positively charged residues Arg234 and Arg235 within the P-stalk pocket of RTA forming a salt bridge with Arg235 (Fig. S6*B*). These interactions are key contacts shared by RU-NT-165, RU-NT-192, and RU-NT-202 (Fig. 5). Arg234 and Arg235 function as anchoring points within the P-stalk pocket, with stronger inhibitors establishing more robust interactions with these residues. Moreover, Leu9 and the aromatic side chain of Phe10 in P11 contribute to hydrophobic interactions with key RTA residues, including Tyr183, Ile247, Ile248, and Phe240 (Fig. S6*B*). Similarly, each P-stalk inhibitor, RU-NT-165, RU-NT-192, and RU-NT-202, establishes comparable hydrophobic interactions within the P-stalk pocket of RTA (Fig. 5). The similar interactions observed between the P11 peptide, and each inhibitor unveil a common mechanism by which these inhibitors target the P-stalk binding site. This shared mechanism suggests that inhibitors effective at disrupting the strongest contacts between the P-stalk proteins and the key residues within the P-stalk pocket of RTA, specifically targeting Arg234 and Arg235, are likely to be more effective in blocking the interaction between the ribosomal P-stalk proteins and RTA, ultimately resulting in more effective toxin inhibition.

The structures of RTA in complex with RU-NT-165, RU-NT-192, and RU-NT-202 revealed that all three compounds bind at the P-stalk binding pocket of RTA similarly. However, different interactions with key P-stalk residues appear to account for their varying inhibitory function. RU-NT-192 demonstrated a 2-fold improvement in the *K*_i_ value compared to RU-NT-165 and a 4-fold improvement in the *K*_i_ value compared to RU-NT-202. The slightly higher potency of RU-NT-192 over RU-NT-165 appears to be partially due to differences in their carboxylate groups. In RU-NT-192, the carboxylate is rotated by 26° relative to the thiophene ring, resulting in reduced conjugation and increased electron density on the carboxylate compared to RU-NT-165 (Fig. 6*B*). This conceivably strengthens the noncovalent interactions between the carboxylate group of RU-NT-192 with Arg234 and Arg235 in RTA. In contrast, RU-NT-165 has a nearly coplanar orientation between the thiophene ring and carboxylate group (Fig. 6*B*), which diminishes the relative strength of its noncovalent interactions with Arg234 and Arg235 compared to RU-NT-192. Unlike RU-NT-165, RU-NT-192 formed a π-π stacking interaction between its benzene ring and Phe240 in RTA (Fig. 5*C*), further enhancing RU-NT-192's interaction with RTA relative to RU-NT-165 and ultimately supporting its higher inhibitory activity. The fluorine atom in the fluorobenzene ring of RU-NT-202 interacted specifically with several nonpolar residues lining the P-stalk pocket (Fig. 5*A*), causing a shift in the position of RU-NT-202's benzene ring within the pocket compared to RU-NT-192, repositioning the thiophene ring and carboxylate group of RU-NT-202 away from Arg234 (Fig. 6*C*). The RU-NT-202 carboxylate also rotated by 91° relative to RU-NT-192, further distancing the carboxylate of RU-NT-202 from Arg234 (Fig. 6*A*). Consequently, the carboxylate group of RU-NT-202 did not interact noncovalently with Arg234, potentially reducing the binding affinity and inhibitory potency compared to RU-NT-192. Furthermore, the relatively altered position of the fluorobenzene ring in RU-NT-202 prevented hydrophobic contacts with Leu207, which may have further diminished the inhibitory function.

**Figure 9:**
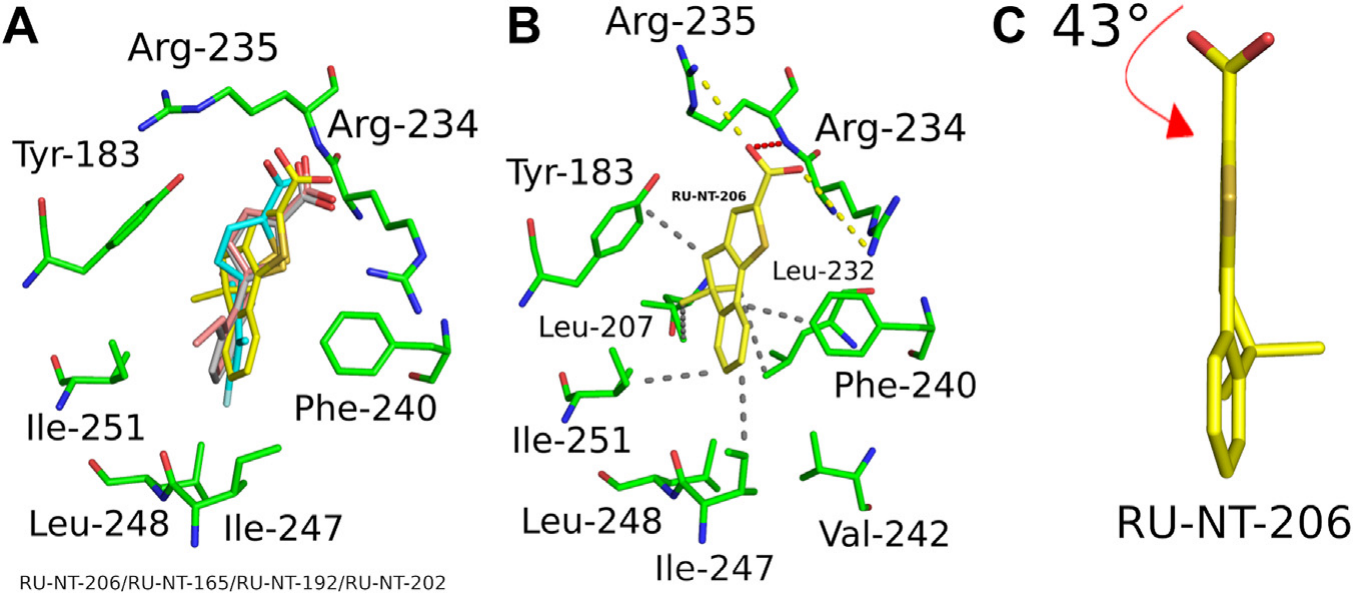
**Slight deviations in the binding mode of each RTA-inhibitor complex.***A*, the comparable binding mode of RU-NT-165 (*gray*), RU-NT-192 (*salmon red*), RU-NT-202 (*cyan*), and RU-NT-206 (*yellow*) with RTA (*green*) disclosed by the superposition of each RTA-inhibitor complex. *B*, close-up of the noncovalent interactions of RTA (*green*) in complex with RU-NT-206 (*yellow*). The salt-bridges and H-bond are represented as *yellow* and *red dashes*, respectively. The nonpolar contacts between RU-NT-206 and RTA are drawn as *gray dashes*. *C*, RU-NT-206 (*yellow sticks*) depicting the relative orientation of its carboxylate relative to the thiophene ring in this inhibitor where 43° angle in RU-NT-206 greatly boosts the electron density around the carboxylate of RU-NT-206. The *red arrow* highlights the rotation of the carboxylate group relative to the thiophene ring in each inhibitor. All nitrogen atoms were colored *blue*, all oxygen atoms were colored *red*, and all sulfur atoms were colored *yellow*.

The crystal structures of RTA alone (PDB ID: 1RTC) and RTA bound to RU-NT-206 (PDB ID: 8TAD) exhibit high similarity to the RTA structures reported here. RU-NT-165, RU-NT-192, and RU-NT-202 bind the P-stalk pocket of RTA similarly to RU-NT-206 (Fig. 9*A*). RU-NT-206 had a ∼6-fold lower EC_50_ value than RU-NT-202, a 3-fold lower EC_50_ value than RU-NT-165, and a similar EC_50_ compared to RU-NT-192 (Table 1). The enhanced potency of RU-NT-206 can be significantly attributed to its thiophene carboxylate, which forms salt bridges with the side chains of Arg234 and Arg235 and a hydrogen bond with the main chain amide nitrogen of Arg235 (Fig. 9*B*). Furthermore, RU-NT-206's cyclohexadiene and benzene rings engage hydrophobically with Tyr183, Leu232, Phe240, Ile247, and Ile251, while the methyl group at the C5 position of the cyclohexadiene ring adds an additional hydrophobic interaction with Leu207 in RTA. The carboxylate group of RU-NT-206 was also rotated by a 43° angle relative to the thiophene ring, boosting the electron density around the carboxylate and supporting superior RTA binding (Fig. 9*C*). RU-NT-206 more closely positioned its carboxylate group at 2.9 Å and 3.8 Å from Arg234 and Arg235, respectively. This proximity allowed the thiophene carboxylate to form salt bridges with the side chains of both Arg234 and Arg235, along with a main chain H-bond with Arg235, which likely increased the binding affinity for RTA and improved its potency in the cell-based assay. These results indicate that interactions of the phenyl group with the nonpolar region of the P-stalk pocket enhance the binding affinity of each inhibitor and influence orientation of the carboxylic acid within the P-stalk pocket, either promoting or hindering crucial contacts with Arg234 and Arg235, which are essential for P-stalk binding and optimal inhibitory potency (57).

We previously showed that RU-NT-206 inhibited ribosome depurination mediated by Stx2A1 at a lower level than RTA. We examined the effect of RU-NT-192 on the depurination activity of Stx2A1 using rat liver ribosomes. RU-NT-192 protected rat liver ribosomes from depurination by Stx2A1 with an IC_50_ value of 61.5 ± 5.4 μM (Fig. S7) using the Hill model with a Hill coefficient (n) of 2.2. The level of protection was similar to the protection of Stx2A1-mediated depurination of rat liver ribosomes by RU-NT-206, which had an IC_50_ value of 62 ± 0.7 μM using the Hill model and a Hill coefficient of 4.1 (Fig. S7). The Hill coefficients were greater than one, suggesting some nonspecific interaction between each compound and Stx2A1. The lower potency of RU-NT-192 and RU-NT-206 against Stx2A1 may be due to the differences in the P-stalk pockets of Stx2A1 and RTA (58) and because these compounds are optimized for the P-stalk site of RTA.

The current understanding of RTA's depurination mechanism has been based exclusively on biochemical methods and X-ray structure analysis. The mechanism by which P-stalk binding stimulates the depurination activity of RTA by ∼200-fold is not known (26, 27). Recent cryo-EM structure of Stx2a with the eukaryotic P-stalk pentamer revealed significant conformational heterogeneity at the C-terminal P-stalk binding site of Stx2a (59). The structural similarities between the RIPs including Stx2A1 and RTA suggests that conformational flexibility in the P-stalk site, which is not apparent from the X-ray structures, could have a profound impact on the specificity of inhibitors and allosteric mediation of function.

Solution NMR is an established technique to probe local and allosteric structural and dynamic changes triggered by ligand binding in proteins (45, 46). Here using CSP analysis, we probed the binding-induced conformational changes in WT RTA complexed with RU-NT-192 and RU-NT-206. By comparing the chemical shift changes, we concluded that the binding mode of both inhibitors is very similar and consistent with the X-ray structure of the inhibitor-bound complexes. A key finding by NMR is that the binding-induced structural rearrangement has a pronounced effect on the CTD of RTA with few changes detected in the N-terminal domain. These results indicate that the CTD of RTA is relatively malleable, and the predominantly hydrophobic-binding pocket can remold itself to anchor different inhibitors or the P11 peptide. By mapping the CSP data on the protein structure (Figs. 7 and 8), we showed that the structural rearrangement in the P-stalk site also influences residues located at the outer surface of the α^10^-helix (202–213), which are implicated in binding the adenine in the rRNA substrate. Notably, mutations at Asn209, Trp211, and Arg213 have a debilitating effect on the enzyme activity (49, 50, 51, 52, 53). These residues all exhibit variable chemical shift changes in the inhibitor complexes. These observations agree with previous studies of RTA bound to the P11 peptide where the active site cleft senses the changes in the CTD (47). Presently the strategic location of the α^10^-helix at the domain interface and its role in coupling the active site with the P-stalk site is poorly understood. In this study, we present promising new evidence, which suggests that a path for allosteric communication exists between these two sites. In future biochemical and NMR experiments, we will evaluate the impact of P-stalk site inhibition on the structure and dynamics of the active site of RTA to parse its contribution to the IC_50_ values.

In this study, we identified compounds that inhibit the cytotoxicity of ricin at low micromolar concentrations with no apparent cellular toxicity by binding the P-stalk site of RTA, indicating that this strategy holds great promise for combatting ricin. We provide the first evidence for the conformational flexibility of the CTD of RTA and its potential role in mediating the catalytic activity of ricin. These results suggest that the inhibitory function of the small molecules is due not only to blocking the interaction of RTA with the ribosomal P-stalk but may also be due to allosteric effects at the active site. This work provides new insight into the P-stalk binding mechanisms that can be exploited in the rational design of more effective compounds.

## Experimental procedures

### Chemistry

**Figure 10:**
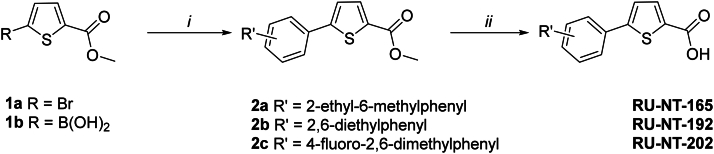
**Synthesis of RU-NT165, RU-NT192, and RU-NT-202.***i* – boronic acid, aryl halogen, Pd(PPh_3_)_4_, K_3_PO_4_, dioxane/water; *ii* – NaOH, THF/water, alternatively LiOH, THF/water/methanol.

CC10501 was purchased from Maybridge, Inc as previously reported (37). Synthesis and the analysis of RU-NT-93 and RU-NT-102 were previously reported (39). RU-NT-206 was purchased from Enamine, Inc. as previously reported (38). RU-NT-202, RU-NT-165, and RU-NT-192 were synthesized by previously reported procedures (39), starting from methyl 5-bromothiophene-2-carboxylate (2a) or, alternatively, where boronic acid was not readily available (2b, 2c), from (5-(methoxycarbonyl)thiophen-2-yl)boronic acid (Fig. 10).

LC-MS and HPLC analyses were performed using the Shimadzu LCMS-2020 system with Dual Ionization Source: 5 μl injection on an XBridge C18 (3.5 μM, 4.6 × 50 mm) column at a temperature of 40 °C with a 4 min gradient from 5% A to 95% B (acidic method: solvent A: 0.1% v/v TFA in water, solvent B: acetonitrile, column at a temperature of 40 °C with a 4 min gradient from 5% A to 95% B) at a flow rate of 2 ml/min. The detection used a diode array scanning from 190 to 600 nm or dual-wavelength detectors at 220 and 254 nm (mass detection cone voltage: 30 V).

NMR was obtained on a Bruker Avance Neo 400 MHz in DMSO-*d*_6_ (1H: δ2.50). Flash column chromatography purifications were performed using Biotage Isolera and Selekt systems. Preparative HPLC was performed on ACCQPrep HP125 system, (Waters XBridge BEH C18 column, 100 × 30 mm × 10 μm; mobile phase: A: 0.1% v/v formic acid in water; B: acetonitrile; 10% to 100%, 14 min).

The HRMS analyses were performed using Bruker Apex 7T FTMS, using an ESI ion source in positive mode. Alternatively, experiments were performed using a Xevo G2-XS qTOF mass spectrometer equipped with an Acquity UPLC system. The UPLC-MS system and the column were from Waters Inc. For the LC separation, the following two eluents were used: A containing 0.1% v/v formic acid in water and B containing neat acetonitrile. Linear gradient (5–100% B in 3 min) was applied. The ionization method was ESI, generating [M-H]^+^, M^+^, or [M+H]^+^ ions as noted in each case. The mass spectrometer was calibrated using a Leu-enkephalin standard. Capillary exit voltage is 330 V. An Agilent 6546 qTOF mass spectrometer equipped with an Agilent Infinity 1290 UPLC was used for further HRMS analyses. For the latter UPLC, the following two eluents were used: A containing 0.1% v/v formic acid in water and B containing 0.1% v/v formic acid in acetonitrile. Linear gradient (5–100% B in 4 min) was applied. ESI ionization generated M^+^, [M+H]^+^, [M+Na]^+^, or [M-H]^-^ ions as noted in HRMS reports. The mass spectrometer was calibrated using a standard supplied by Agilent Inc. For the UPLC systems, a 2.1 mm × 50 mm BEH C18 column (particle size 1.7 μm) was utilized. Unless otherwise stated, the purities of the final compounds were equal to or greater than 95% by HPLC analysis. The purities were confirmed with ^1^H NMR to look for residual solvents or non-UV active impurities.

5-(4-fluoro-2,6-dimethylphenyl)thiophene-2-carboxylic acid (RU-NT-202) has been prepared according to a previously reported procedure (39). ^1^H NMR: (500 MHz, DMSO-*d6*): δ 7.60 (d, *J* = 3.7 Hz, 1H), 6.80 (d, *J* = 3.6 Hz, 1H), 6.32 (s, 2H), 1.95 (s, 6H).

HRMS: [M+H]^+^ calculated 251.0537, found 251.0536, error −0.40 ppm.

5-(2-Ethyl-6-methylphenyl)thiophene-2-carboxylic acid (RU-NT-165) has been prepared according to a previously reported procedure (39). ^1^H NMR: (500 MHz, CDCl_3_): δ 7.92 (d, *J* = 3.6 Hz, 1H), 7.28 (t, *J* = 7.6 Hz, 1H), 7.15 (dd, *J* = 19.1, 7.6 Hz, 2H), 6.89 (d, *J* = 3.8 Hz, 1H), 2.48 (q, *J* = 7.6 Hz, 2H), 2.15 (s, 3H), 1.12 (t, *J* = 7.5 Hz, 3H).

HRMS: [M-H]^-^ calculated: 245.0642, found 245.0635, error: −2.73.

5-(2,6-Diethylphenyl)thiophene-2-carboxylic acid (RU-NT-192) has been prepared according to a previously reported procedure (39). ^1^H NMR: (500 MHz, DMSO-*d6*) δ 13.14 (s, 1H), 7.76 (d, *J* = 2.9 Hz, 1H), 7.34 (t, *J* = 7.8 Hz, 1H), 7.18 (d, *J* = 7.8 Hz, 2H), 7.02 (t, *J* = 3.0 Hz, 1H), 2.37 (q, *J* = 7.4, 2.0 Hz, 4H), 1.04 (t, *J* = 8.8 Hz, 6H).

HRMS: [M-H]^-^ calc. 259.07982, found 259.0810, error: 4.55 ppm.

### *In vitro* depurination inhibition

*In vitro* depuration inhibition assay was carried out as published (37, 38, 39). In brief, RTA (200 pM) was mixed with different concentrations of compounds in the depurination buffer (20 mM Hepes pH 7.5, 25 mM KCl, and 5 mM MgCl_2_), and rat liver ribosomes were added to start the reaction. The reaction was set at room temperature for 5 min, which is in the linear range of the depurination reaction. The reaction was stopped by adding an equal amount of 2× RNA extraction buffer (50 mM Tris–HCl pH 8.8, 240 mM NaCl, 20 mM EDTA, and 2% SDS), and RNA was extracted and the depurination level was measured by qRT-PCR (42). A reaction without RTA and compound was set as no depurination control and a reaction with RTA but no compound was set as 100% depurination control for each experiment. The experiment was repeated 2 to 4 times. The IC_50_ was calculated by fitting the percent inhibition data using the Hill equation (44, 54).

$$y=Vmax\frac{x^{n}}{k^{n}+x^{n}}$$

where y = percent inhibition, V_max_ = maximum inhibition, x = concentration of inhibitor, k = IC_50_ and n = Hill coefficient.

### Mammalian cell culture and treatment for depurination analysis

Vero cells (ATCC #CCL-81) were cultured and maintained in 250 ml Falcon flasks (VWR #353136). Vero cells were determined to be free of *mycoplasma* contamination for all experiments. Cells were grown in Dulbecco's Modified Eagle Medium (DMEM), containing glutamine (Gibco, #11995-065), penicillin and streptomycin (Gibco Pen Strep #15140122), plus fetal bovine serum (FBS, Gibco #A5209401). Cell flasks were kept in an incubator maintained at 37 °C, 5% CO_2_, and Class 100 HEPA air filtration (Thermo Scientific Series 8000 Water-Jacketed CO_2_ Incubator). At 24 h prior to depurination testing, cells were detached from the plate with a 5-min trypsin treatment (Gibco # 25200056) and counted by flow cytometry (BD Life Sciences Accuri C6 Plus), resuspended in DMEM (Pen Strep + FBS) at a density of 1.5 × 10^5^ cells/ml, and 500 μl of cells/well were added to labeled 24-well tissue culture plates (VWR Cat. #10062-896). After 24 h, the media was removed and cells were treated with a dilution series of the compound to be tested, prepared in DMEM (Pen Strep-FBS) with ricin. Final ricin concentration tested was 200 pM. Final compound concentrations ranged from 500 μM to 15.6 μM, and a constant 0.5% dimethyl sulfoxide (DMSO, Sigma D2650) was maintained across treatments. A control group treated with toxin alone (200 pM ricin) and a vehicle control group treated with 0.5% DMSO were included. Cells were incubated with the compound for 2 h at 37 °C. After incubation, the media was removed, and the cells were lysed with 350 μl of RLT Plus buffer (Qiagen RNeasy Plus Mini Kit #74136) containing 0.1% β-mercaptoethanol. The plates were then wrapped in aluminum foil and stored at −80 °C for subsequent RNA isolation.

### RNA isolation and depurination inhibition in mammalian cells

Total RNA was extracted from thawed cell lysates using the RNeasy Plus Mini Kit (Qiagen, Cat. #74136) with minor modifications to the spin times outlined in the manufacturer's protocol. Briefly, 350 μl of cell lysate was processed through the gDNA Eliminator column with a 30-s centrifugation step. Following the 70% ethanol addition, the mixture was transferred to an RNeasy spin column and centrifuged for 20 s. The column was washed with 700 μl RW1 buffer (30-s spin) and two washes with 500 μl RPE buffer (30-s and 2-min spins, respectively). After a 1-min drying spin, RNA was eluted in 30 μl of RNase-free water. All centrifugation steps were performed at room temperature at maximum speed. RNA concentration was quantified using an Agilent BioTek Synergy H1 microplate reader with Gen5 software.

The cDNA synthesis was performed using the High-Capacity cDNA Reverse Transcription Kit (Applied Biosystems, Thermo Fisher Scientific, #4368814) with 350 ng of input RNA per reaction. A master mix containing 10× RT buffer, 10× random primers, 100 mM dNTPs, and MultiScribe reverse transcriptase was prepared in RNase-free water. For RNA concentrations ≥170 ng/μl, 2 μl of RNA was used per reaction. Reverse transcription was carried out in an Applied Biosystems GeneAmp PCR System 9700 with the following thermal cycling conditions: 10 min at 25 °C, 2 h at 37 °C, and 5 min at 85 °C. cDNA was stored at −20 °C until further use.

qRT-PCR was conducted on an Applied Biosystems StepOne Plus instrument using StepOne software v2.3. Reactions were performed in triplicate with 2× Power SYBR Green PCR Master Mix (Applied Biosystems, Thermo Fisher Scientific, #4367659) and 100 μM of each sequence-specific primer. Forward and reverse primers used were as follows: 28S rRNA, 5′-GATGTCGGCTCTTCCTATCATTGT-3′ and 5′-CCAGCTCACGTTCCCTATTAGTC-3′; Depurinated rRNA, 5′-TGCCATGGTAATCCTGCTCAGTA-3′ and 5′-TCTGAACCTGCGGTTCCACA-3′. A 1:50 dilution of cDNA was used as a template. The following cycling conditions were employed: 95 °C for 10 min, followed by 40 cycles of 95 °C for 15 s and 60 °C for 60 s. A melt curve analysis was performed to assess amplicon specificity. The relative abundance of depurinated rRNA was determined using the comparative C_T_ method (ΔΔC_T_) with 28S rRNA as the endogenous control. The EC_50_ was calculated by fitting the percent inhibition data using the Hill equation (44, 54).

### Mammalian cell viability assay

Cell viability was assessed using Cell Titer Glo-3D reagent (Promega, #G9682). For compound screening, 100 μl of mammalian cells (1.5 × 10^5^ cells/ml) were added to each well of a sterile, white tissue culture–treated 96-well plate (Corning #3917) and allowed to grow for 24 h. After 24 h, the media was removed from the wells and replaced with 100 μl of media containing varying concentrations of compound and ricin. Compounds were prepared by diluting them in serum-free DMEM media (Gibco, #11960044) and DMSO (Sigma, #D2650) to achieve 2× working concentrations. Compounds were screened at 500 μM, 250 μM, 125 μM, and 0 μM final concentrations. Ricin (Vector Labs) was diluted in DMEM media minus serum to a 2× working concentration of 4000 pM and serially diluted 1:3 to generate eight concentrations ranging from 0 to 2000 pM. Fifty five microliters of compounds at 2× working concentration were then mixed with 55 μl of each 2× ricin concentration in a separate, sterile, non-tissue culture–treated 96-well plate (Corning, #351172). The final 1× compound/ricin mixture (100 μl) was then added to the corresponding wells of the 96-well plate containing the mammalian cells. The final concentration of DMSO was 0.1% in all wells. The plates were incubated for 48 h at 37 °C. The plates were then removed from the incubator and allowed to equilibrate to room temperature for 15 min. Cell Titer Glo-3D reagent (100 μl) was added to each well, mixed by pipetting up and down several times, and the plates were shaken for 5 min. The plates were then incubated at room temperature for 15 min protected from light. Luminescence was measured using a BioTek plate reader. Data were analyzed using the OriginPro 2023 software.

### Cloning and protein production for crystallization

RTA was cloned and expressed as previously described (38).

### Crystallization and data collection

For crystallization trials, RTA was concentrated to 10 mg/ml and incubated with 2 mM of each inhibitor for 30 min at room temperature before setting up the crystallization experiments. Crystals of the RTA-inhibitor complexes with RU-NT-165 and RU-NT-192 were grown using the sitting drop vapor diffusion method at 20 °C while RTA-RU-NT-202 grew at 4 °C, with a protein-to-reservoir volume ratio of 1:1 and a total drop volume of 0.2 μl. The crystallization conditions are listed in Table S1. Afterward, crystals were flash-frozen in liquid nitrogen following a brief soak in the corresponding crystallization buffers, supplemented with 25% ethylene glycol for the RTA-RU-NT-165 and RTA-RU-NT-192 crystals and 25% sucrose for RTA-RU-NT-202 crystals. Data were collected at the 24-ID-E beamline at the Advanced Photon Light Source, Argonne National Labs. All data was indexed, merged, and scaled using HKL2000, then converted to structure factors using CCP4 7.0.

### Structure determination and refinement

Each RTA-inhibitor complex structure was solved using molecular replacement, with the RTA coordinates (PDB ID: 1RTC) serving as the search model for all complexes. The phase information obtained from molecular replacement was then used to fit and position each fragment inhibitor into the corresponding electron density omit maps using the molecular graphics software COOT 8.9.2. The electron density for the ligands bound to RTA was clearly defined in the initial difference density maps. The structures of RTA-RU-NT-165, RTA-RU-NT-192, and RTA-RU-NT-202 were all solved at 1.8 Å with RTA-RU-NT-165, RTA-RU-NT-192 in the P2_1_ space group and RTA-RU-NT-202 in the P2_1_2_1_2_1_ space group. The isomorphous RTA-RU-NT-165 and RTA-RU-NT-192 crystals had two copies of RTA per asymmetric unit while the RTA-RU-NT-202 crystals had one copy of RTA in the asymmetric unit. Each copy of RTA within the asymmetric unit from the RTA-RU-NT-165 and RTA-RU-NT-192 complexes were very similar with an RMSD ranging from 0.2 to 0.3 Å. Structural refinement of all coordinates was carried out using the PHENIX 1.20.1 package. A cross-validation test set was generated by randomly selecting 5% of the reflections. Data collection and refinement statistics are provided in Table S2. Molecular graphics were created with PyMOL 4.6 (Schrodinger) (DeLano Scientific LLC). Initially, each fragment inhibitor was omitted from the model during the first stages of refinement. After several cycles, each fragment inhibitor was fitted into the corresponding electron density and refined to convergence. The final refinement statistics are summarized in Table S2. B-factor analysis was conducted using the BAVERAGE program from the CCP4 7.0 suite, and additional structural analysis was performed using the virtual reality software, Nanome (60).

### NMR spectroscopy

The NMR data were acquired on Bruker *AVANCE* III spectrometers equipped with TCI CryoProbes at 16.4T with isotopically enriched protein samples prepared using established protocols (47). The previously published backbone amide (NH) and methyl group (CH_3_) chemical shift assignments of RTA∗ with active site mutants were transferred to the WT protein using a suite of triple-resonance (HNCO, HNCA, (H)CCH-TOCSY) experiments (61) and 3D ^15^N-edited NOESY-HSQC acquired on a U-^13^C/^15^N-labeled RTA (200 μM) at 16.4T. The mixing time for the DIPSI2 sequence is set to 16 milliseconds in (H)CCH-TOCSY and 100 milliseconds in the ^1^H-^1^H NOE experiments.

To map the binding site of the small molecules (RU-NT-192 and RU-NT-206), 200 μl of 100 μM C^13^/N^15^-labeled RTA was mixed with 10 μl of 10 mM compound dissolved in 100% DMSO. The NMR sample buffer included trace protease inhibitors (Roche protease Inhibitor EDTA free tablets), 50 mM Hepes buffer, 150 mM NaCl, 1 mM TCEP in 5% D_2_O/95% H_2_O at pH 7.5. The amide and methyl chemical shifts were measured by acquiring 2D ^1^H-^15^N HSQC and 2D ^1^H-^13^C HMQC spectra at 16.4 T on the free protein and the inhibitor complex at ∼1:5 protein-to-compound ratio. In the inhibitor complex, the backbone amide assignments were confirmed by 3D-HNCO. All the NMR data were processed in Topspin 3.5 from Bruker Biospin, and the spectra analyzed in CARA 1.5.

## Data availability

The structures generated in this study were deposited in the Protein Data Bank (PDB; http://www.rcsb.org/pdb/) under accession number 9E3T for the RTA-RU-NT-165 complex, 9E42 for the RTA-RU-NT-192 complex, and 9E40 for the RTA-RU-NT-202 complex as described in Table S2. Authors will release the atomic coordinates and experimental data upon article publication. The results of the NMR CSP analysis have been deposited in the Mendeley Data repository, doi: 10.17632/nvr6g3fwgb.2.

## Supporting information

This article contains supporting information.

## Conflict of interest

The authors declare that they have no conflicts of interests with the contents of this article.
