# Supplementary material for: Binding of small molecules at the P-stalk site of ricin A subunit trigger conformational changes that extend into the active site
Source: J Biol Chem. 2025 Feb 14;301(3):108310. doi: 10.1016/j.jbc.2025.108310 (PMC11946510; doi:10.1016/j.jbc.2025.108310)

SUPPORTING INFORMATION

Binding of small molecules at the P-stalk site of ricin A subunit trigger conformational changes that extend into the active site

John E. McLaughlin[1ǂ], Michael J. Rudolph[2ǂ], Arkajyoti Dutta[1], Xiao-Ping Li[1], Anastasiia M. Tsymbal[3] , Yang Chen[2], Shibani Bhattacharya[2], Benjamin Algava[1], Michael Goger[2], Jacques Y. Roberge[3] and Nilgun E. Tumer[1*]

From the [1]Department of Plant Biology, Rutgers, The State University of New Jersey, 59 Dudley Road, New Brunswick, NJ 08901, USA

[2]New York Structural Biology Center, 89 Convent Ave, New York, NY 10027, USA

[3]Molecular Design and Synthesis Core, Rutgers University Biomolecular Innovations Cores, Office for Research, Rutgers University, 610 Taylor Rd, Piscataway, NJ, 08854, USA

ǂThese authors contributed equally and share first authorship.

[*]To whom correspondence should be addressed.

Nilgun E. Tumer, Ph.D.

Department of Plant Biology

Rutgers, The State University of New Jersey

59 Dudley Road, New Brunswick, NJ 08901

Tel: 848-932-6359 Fax: 732-932-6535

E-mail: tumer@sebs.rutgers.edu

Supporting Information

Supplementary Table S1. RTA-inhibitor crystallization solutions

Supplementary Table S2. Data collection and refinement statistics of RTA complexes

Figure S1. Fluorescence anisotropy assay to measure displacement of labeled P11 peptide from RTA using small molecules and P11 peptide.

Figure S2. Correlation curve between the K_i values and the Vero cell EC_{50} values.

Figure S3. Final electron density maps for each RTA inhibitor.

Figure S4. Comparable binding mode of each inhibitor with RTA.

Figure S5. Similar structures of RTA bound to different inhibitors.

Figure S6. Binding mode similarity of RTA-inhibitors with P11 peptide.

Figure S7. Inhibition of Stx2A1-mediated depurination of rat liver ribosomes by small molecule inhibitors.

NMR Spectra for RU-NT-165, RU-NT-192 and RU-NT-202

HRMS spectra for RU-NT-165, RU-NT-192 and RU-NT-202

PDB Validation reports for the structures of RTA with RU-NT-165, RU-NT-192 and RU-NT-202

TABLES

Supplementary Table S1

**Table S1. RTA-inhibitor crystallization solutions**	
**Inhibitor**	**Crystallization solutions**
RU-NT-165	100 mM Hepes pH 6.5 and 20% PEG 6000
RU-NT-192	170 mM diammonium hydrogen phosphate, 23% PEG 3350, 10 mM Cadmium Chloride
RU-NT-202	Tris pH 7.0, 40% PEG 300, and 5% PEG 1000

Supplementary Table S2. Data collection and refinement statistics of RTA complexes

**Data Collection**			
Complex	RTA-165	RTA-192	RTA-202
Space group	$P2_1$	$P2_1$	$P2_12_12_1$
Cell parameters: *a,b,c* (Å)	38.9, 57.5, 124.9	38.9, 56.9, 124.9	36.5, 81.7, 89.8
APS Beamline	24-ID-E	24-ID-E	24-ID-E
Resolution range (Å)	50-1.80 (1.83-1.80)	50-1.80 (1.84-1.80)	50-1.80 (1.83-1.80)
wavelength (Å)	0.979	0.979	0.979
No. of reflections	85134	50615	47830
Average redundancy[a]	3.5(3.5)	3.4(3.1)	4.8(3.4)
Wilson B-factor (Å^2)	16.4	24.2	8.6
(*I*)/(δ)[a]	8.7(1.1)	10.7(1.5)	32.5(7.8)
Completeness[a] (%)	95.1(95.9)	99.5(96.5)	99.8(99.7)
$R_{merge}^{a,b}$ (%)	7.5 (75.4)	10.4 (92.0)	6.0(26.3)
$CC_{1/2}^{c}$	(0.49)	(0.61)	(0.95)
**Refinement**			
Bragg spacings (Å)	42.24-1.80	42.05-1.80	39.34-1.80
R^d / R_{free}^e (%)	19.5 / 22.3	22.1 / 24.6	15.8 / 18.0
No. of Protein atoms	4110	4052	2101
No. of Ligand atoms	34	44	17
No. of Waters	571	324	340
RMSD bond length (Å)	0.004	0.003	0.005
RMSD bond angle (°)	0.59	0.59	0.70
Average B-factors Protein atoms (Å^2)	18.3	35.5	14.0

Average B-factors Ligand (Å^2)	19.4	37.9	19.2
Average B-factors Water atoms (Å^2)	30.7	41.1	28.5
Ramachandran favored / allowed[f] (%)	99.4 / 100	99.2 / 100	99.2 / 100
PDB code	9E3T	9E42	9E40

[a] Values in outermost shell are given in parentheses.
[b] $R_{merge} = (\sum | I_i - \langle I_i \rangle |)/\sum | I_i |$, where I_i is the integrated intensity of a given reflection.
[c] $CC^{1/2}=\sqrt{2CC1/2/1+CC1/2}$, where CC1/2 is the correlation coefficient of two split data sets each derived by averaging half of the observations for a given reflection.
[d] $R = \sum | |F_o| - |F_c| | / \sum | |F_o| |$, where F_o and F_c denote observe and calculated structure factors, respectively.
[e] R_{free} was calculated using 5% of data excluded from refinement.
[f] Calculated using Molprobity.

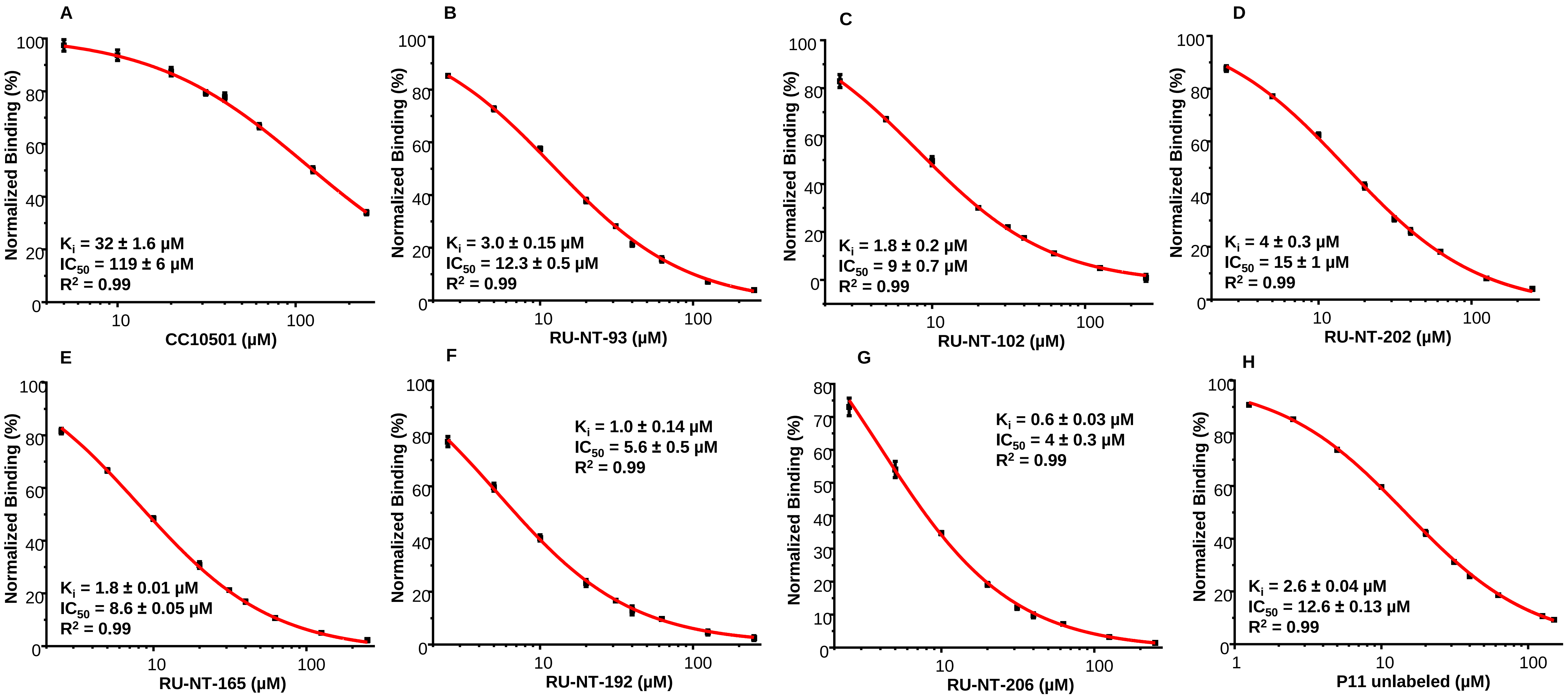

Figure S1. Fluorescence anisotropy assay to measure competitive displacement of fluorescently labeled P11 peptide from RTA using small molecules and unlabeled P11 peptide. Varying concentrations (1.25, 2.5, 5, 10, 20, 31.25, 40, 62.5, 125, and 250 µM) of small molecules (A to G) and unlabeled P11 peptide (H) were incubated with 1 µM BODIPY-TMR labeled P11 peptide and 3 µM of purified RTA in 1X FA reaction buffer (20 mM Tris-Cl pH 7.9 and 100 mM NaCl) for 30 minutes at room temperature. Samples were analyzed using a BioTek Synergy H1 microplate reader. The parallel and perpendicular intensities obtained were used to calculate the IC_{50} and the K_i values. The standard error was determined from four different measurements.

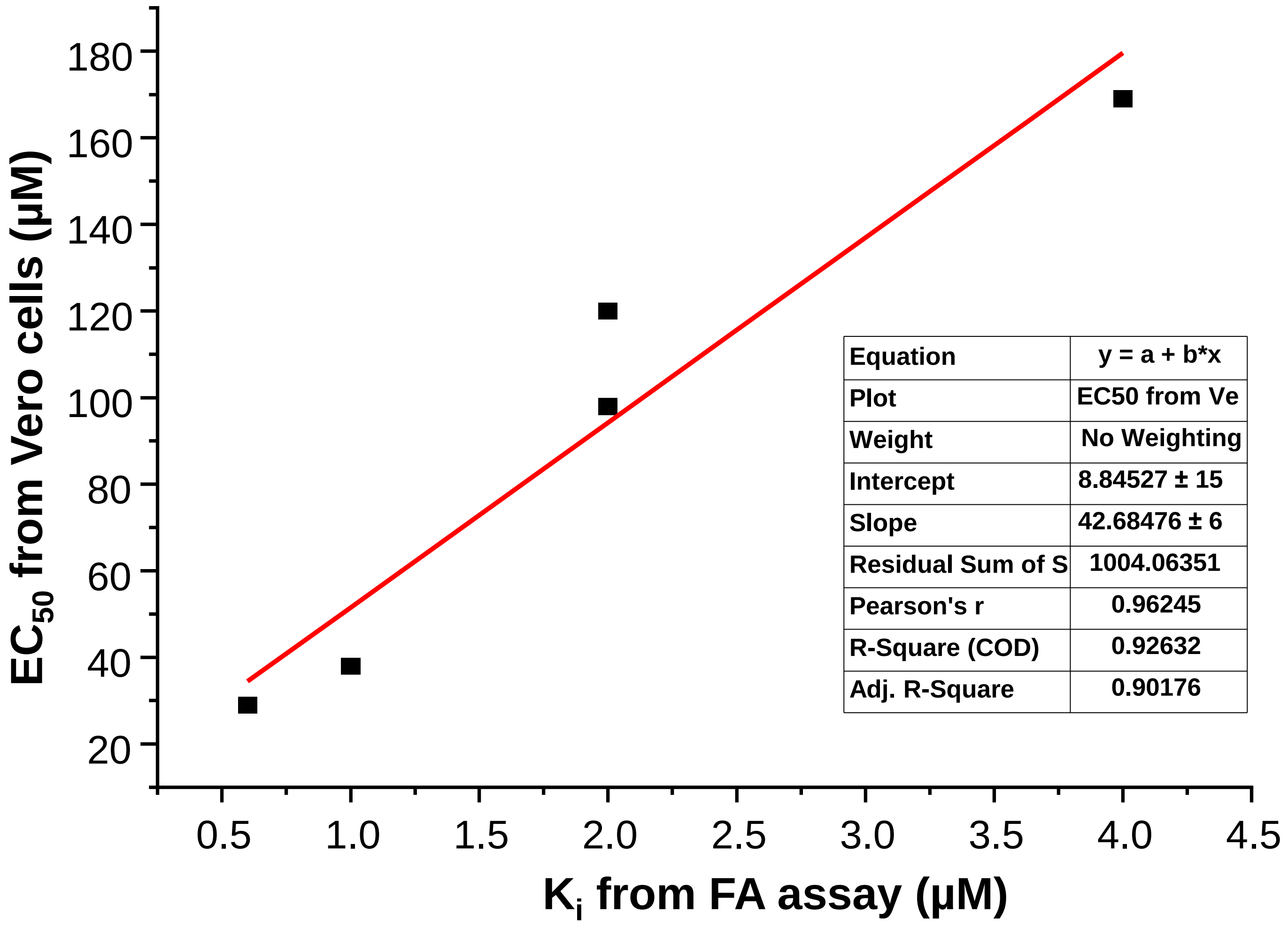

Figure S2. Correlation curve between the K_i values and the Vero cell EC_{50} values. Data for **RU-NT-202**, **RU-NT-102**, **RU-NT-165**, **RU-NT-202** and **RU-NT-206** listed in Table 1 were plotted. Pearson's 'r' value is 0.96 showing that the binding affinities of the compounds for RTA are directly proportional to their inhibitory activity against ricin holotoxin-mediated depurination in Vero cells.

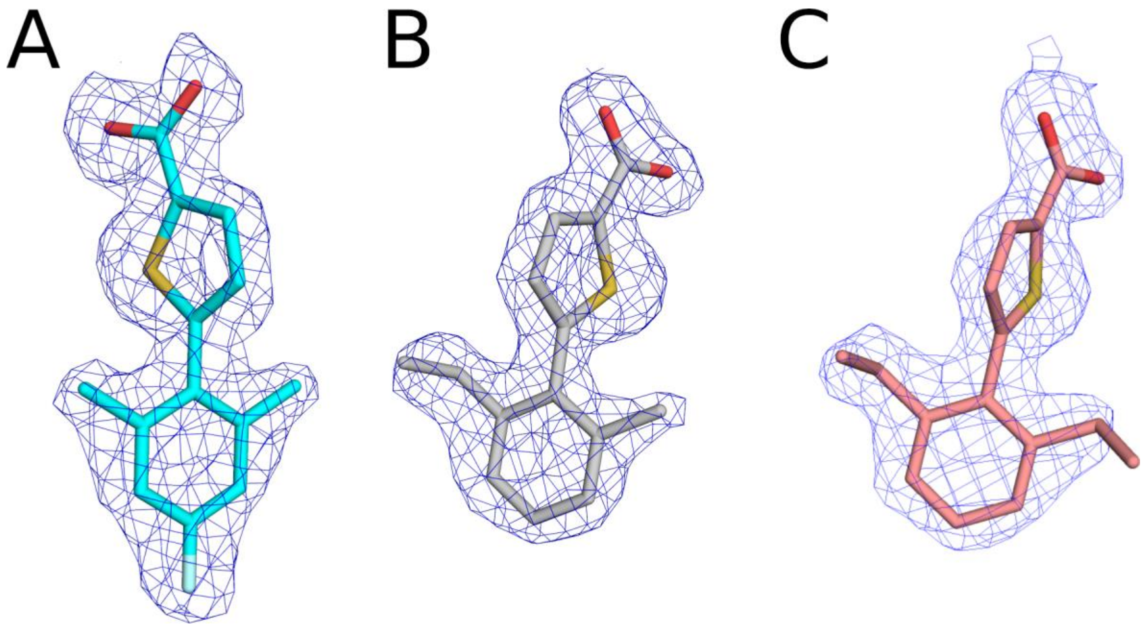

Figure S3. Final electron density maps for each RTA inhibitor. Structure of each inhibitor (A) **RU-NT-202** (cyan), (B) **RU-NT-165** (gray), and (C) **RU-NT-192** (salmon red) drawn as sticks along with the fully refined 2Fo-Fc electron density map (blue mesh) for each inhibitor contoured at a 1.0 σ level. The maps were calculated from fully-converged RTA-inhibitor structures. Each inhibitor is drawn as sticks with all carbon atoms in **RU-NT-202** colored cyan, in **RU-NT-165** colored gray, and in **RU-NT-192** colored salmon red. All oxygen atoms are colored red and sulfur atoms yellow.

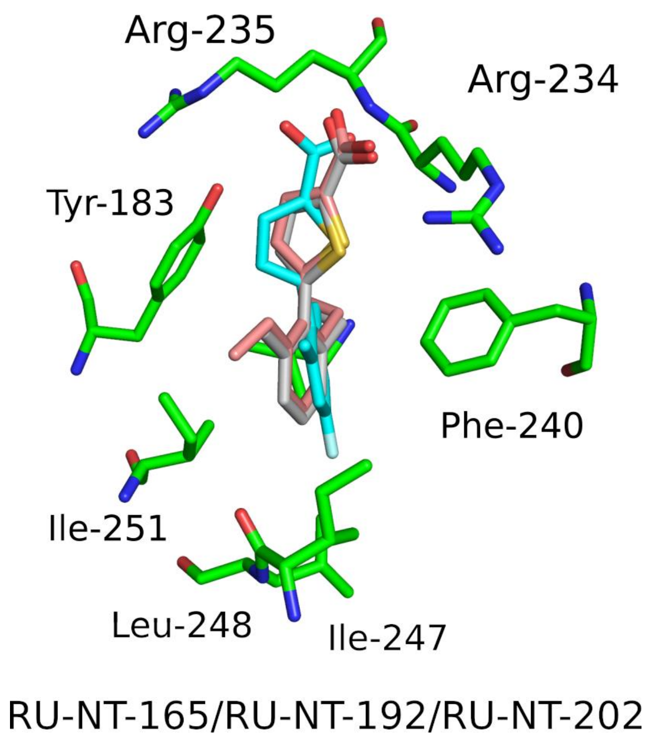

Figure S4. Comparable binding mode of each inhibitor with RTA. The related position of the superposed **RU-NT-165** (gray), **RU-NT-192** (salmon red), and **RU-NT-202** (cyan) bound to the P stalk pocket of RTA (green). All molecules are drawn as sticks with nitrogen atoms colored blue, oxygen atoms colored red, and sulfur atoms colored yellow.

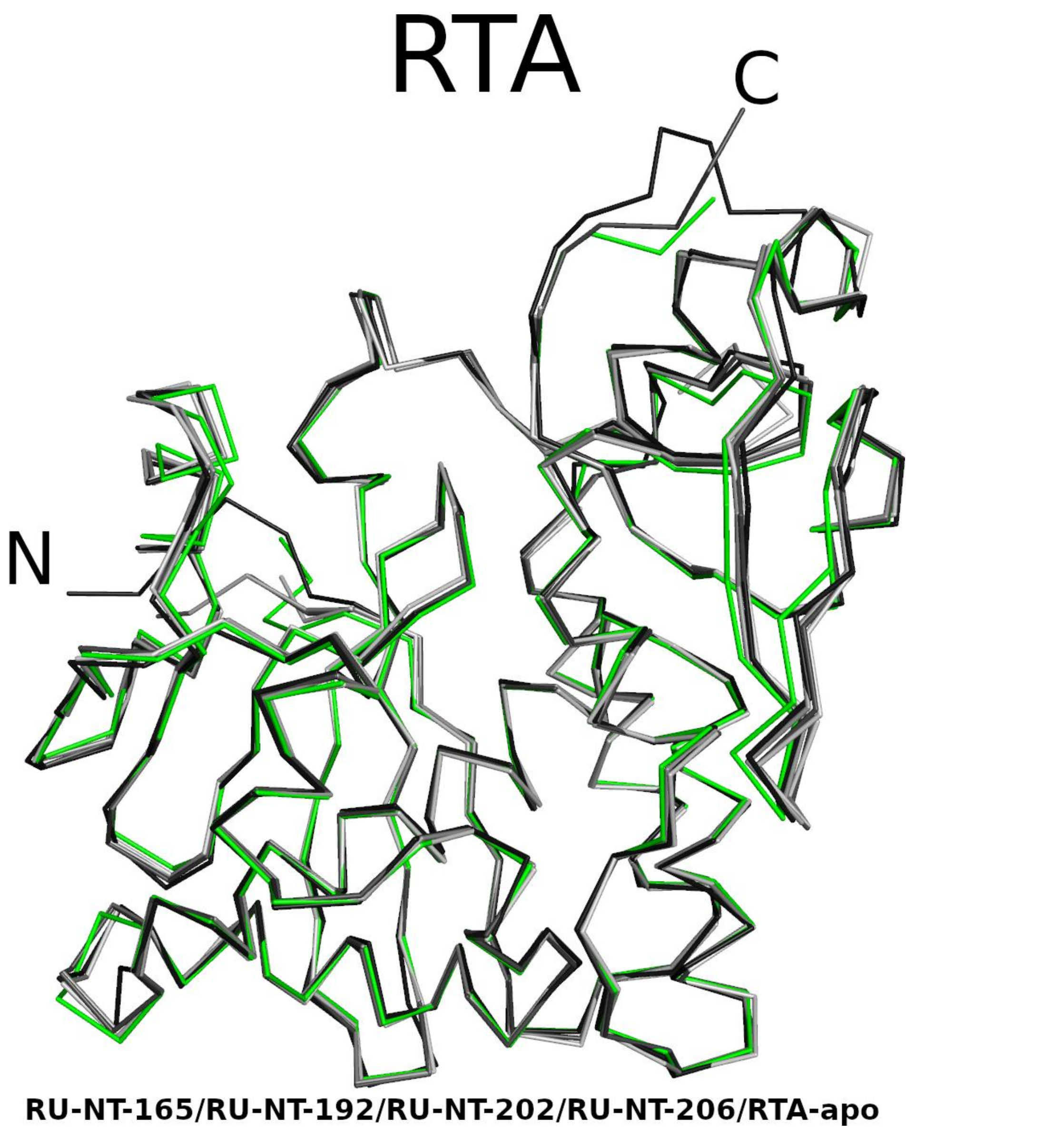

Figure S5. Similar structures of RTA bound to different inhibitors. Shown are the super positioned Cα-traces of RTA when bound to **RU-NT-165**, **RU-NT-192**, **RU-NT-202**, **RU-NT-206**, and in the apo form of RTA colored from dark gray to light gray.

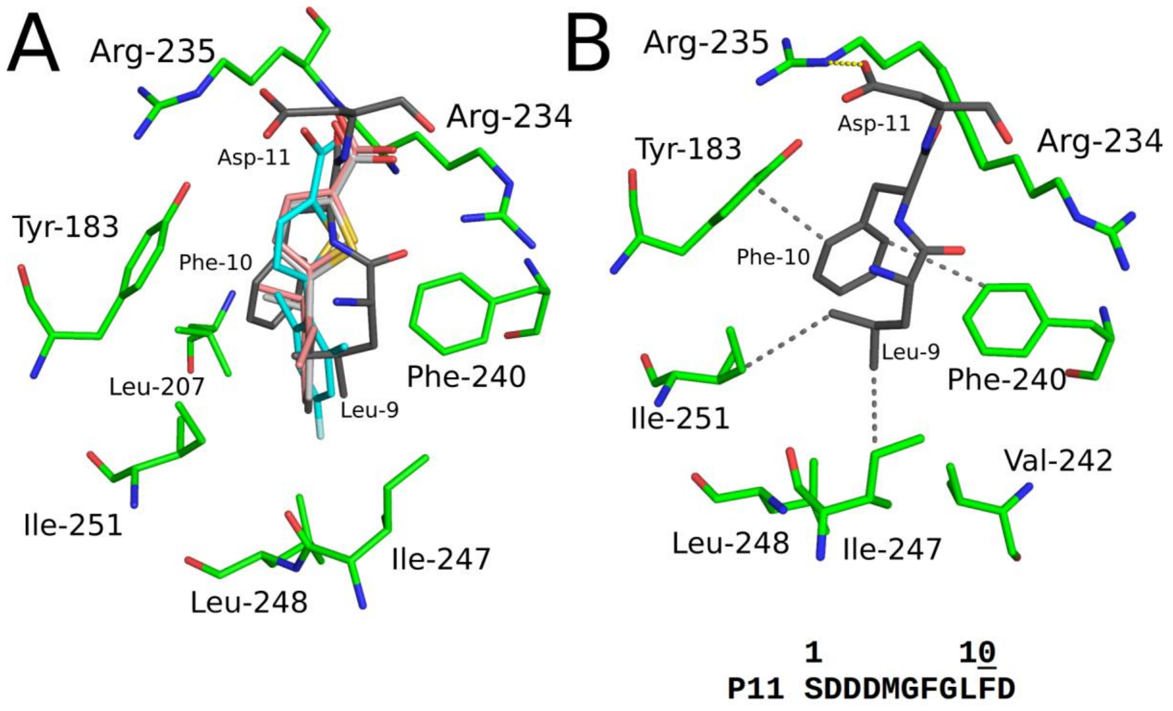

Figure S6. Binding mode similarity of RTA-inhibitors with P11 peptide. (A) The similar Binding mode of RTA (green) with **RU-NT-165** (gray), **RU-NT-192** (salmon red), **RU-NT-202** (cyan), and the P11 peptide (dark gray) is revealed by the superposition of each RTA complex. (B) Close-up of the noncovalent interactions between RTA (green) and the P11 peptide (dark gray). RTA and P11 are all drawn as sticks. The salt bridge is represented by yellow dashes with nonpolar contacts as gray dashes. The P11 primary sequence is depicted in bold text. All molecules are drawn as sticks with nitrogen atoms colored blue, oxygen atoms colored red, and sulfur atoms colored yellow.

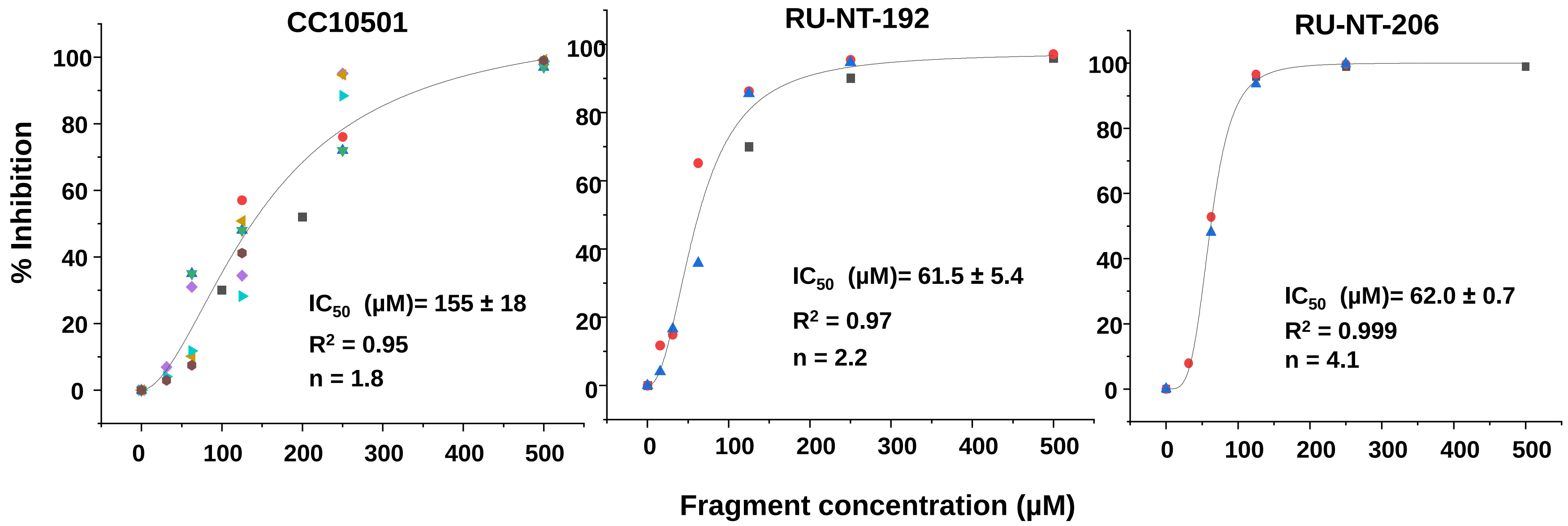

Figure S7. Inhibition of Stx2A1-mediated depurination of rat liver ribosomes by small molecule inhibitors. The experiment was set up same as in Figure 1. Measurements were repeated 2-6 times as indicated by different symbols. The different colored data points represent different biological replicates. The data for the percentage of inhibition at different compound concentrations were fitted with the Hill equation using OriginPro 2023 to calculate the 50% inhibitory activity (IC_{50}). The "n" values represent the Hill coefficient.

RU-NT-165

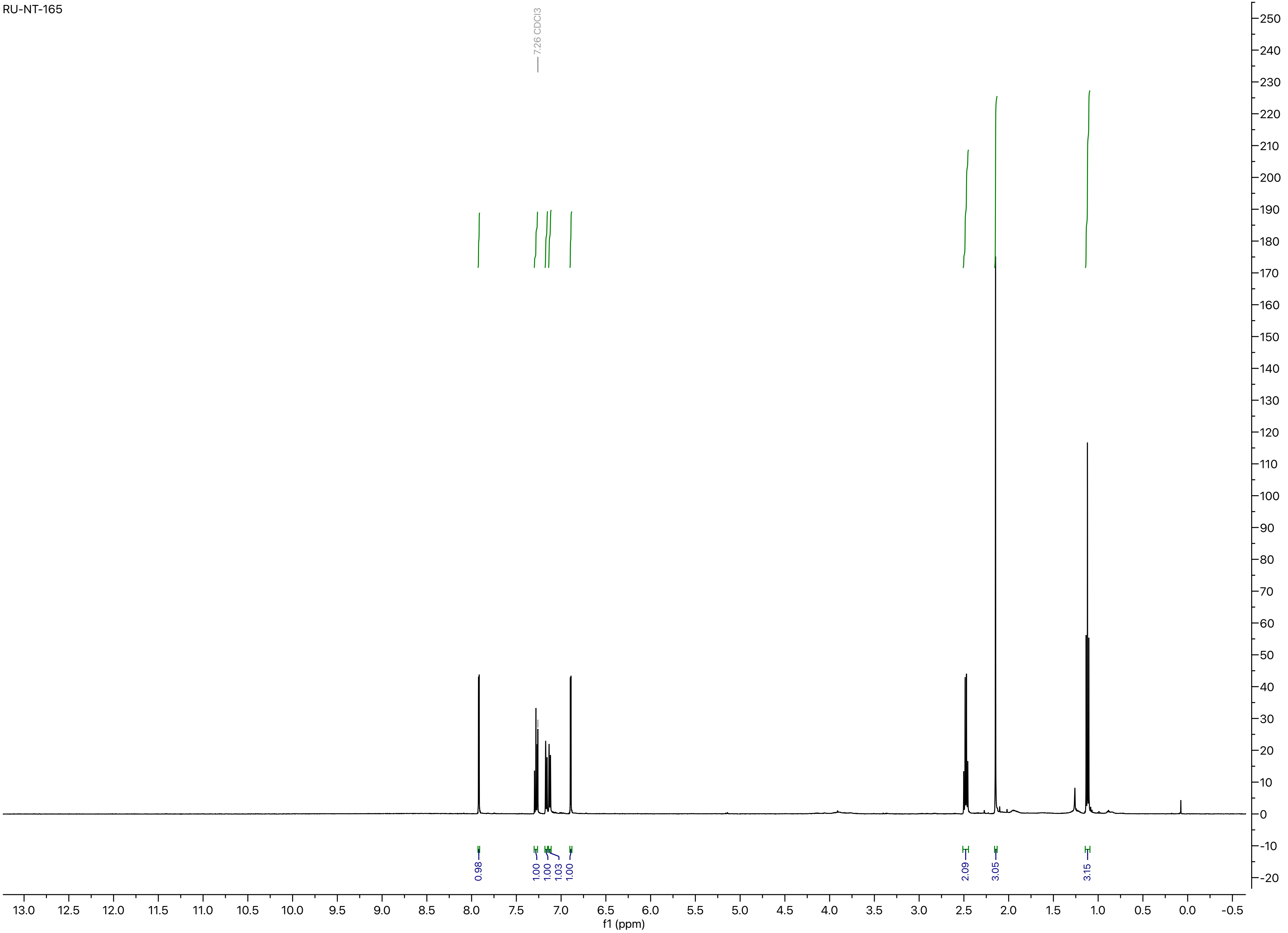

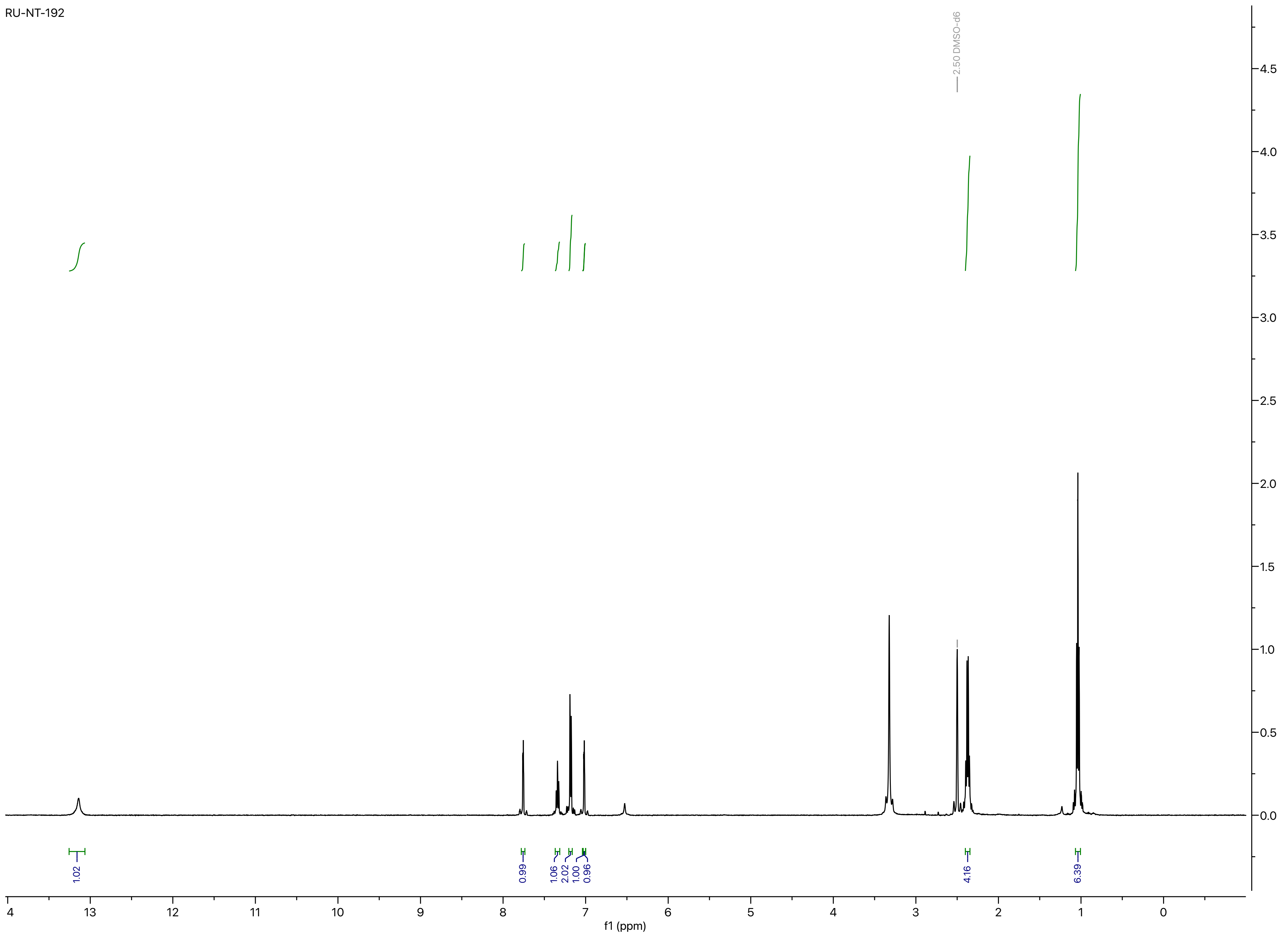

RU-NT-192
2.50 DMSO-d6
1.02
0.99
1.06
2.02
1.00
0.96
4.16
6.39
f1 (ppm)

RU-NT-202

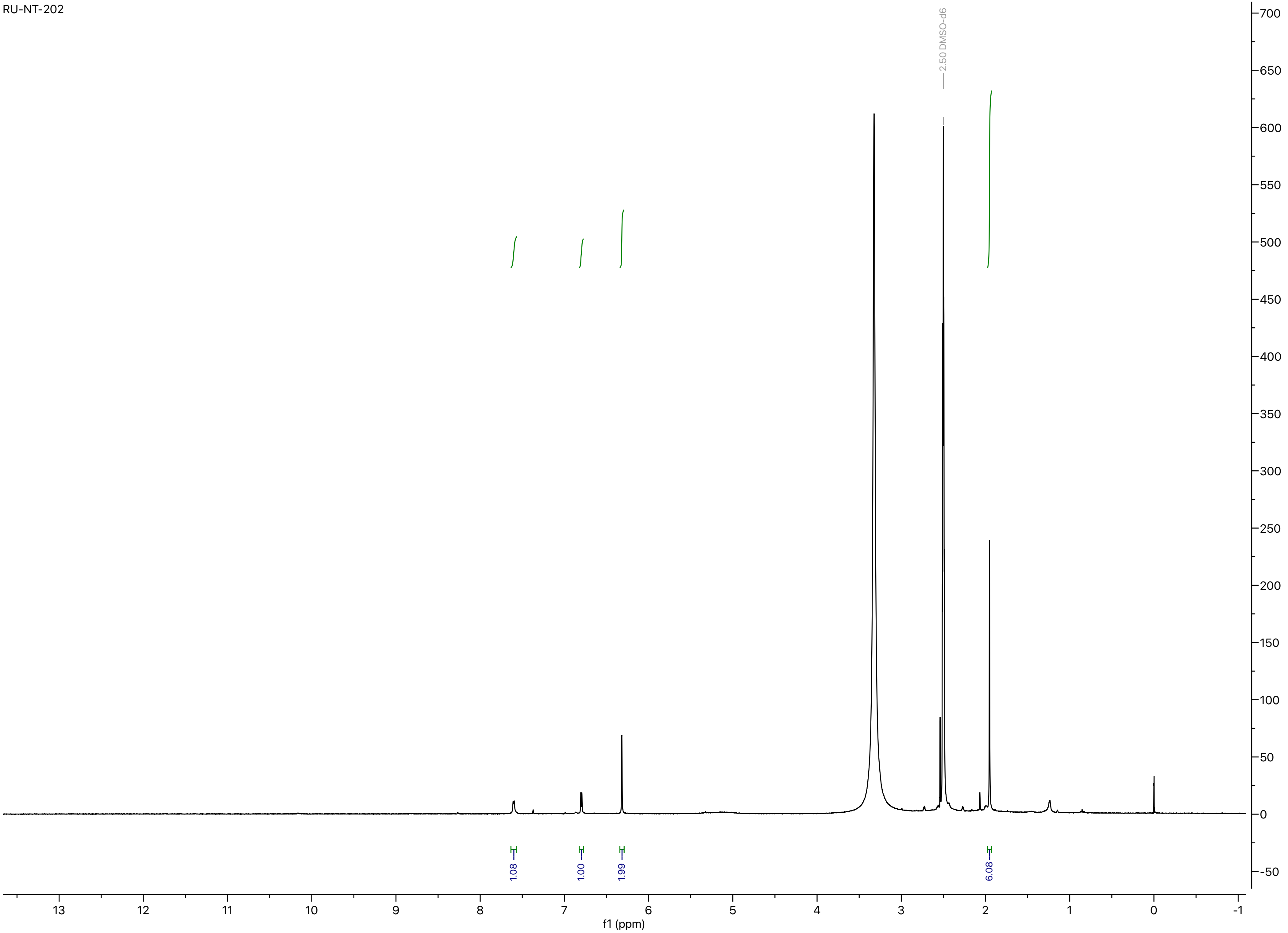

RU-NT-165 HRMS

User Spectrum Plot Report

Agilent Trusted Answers

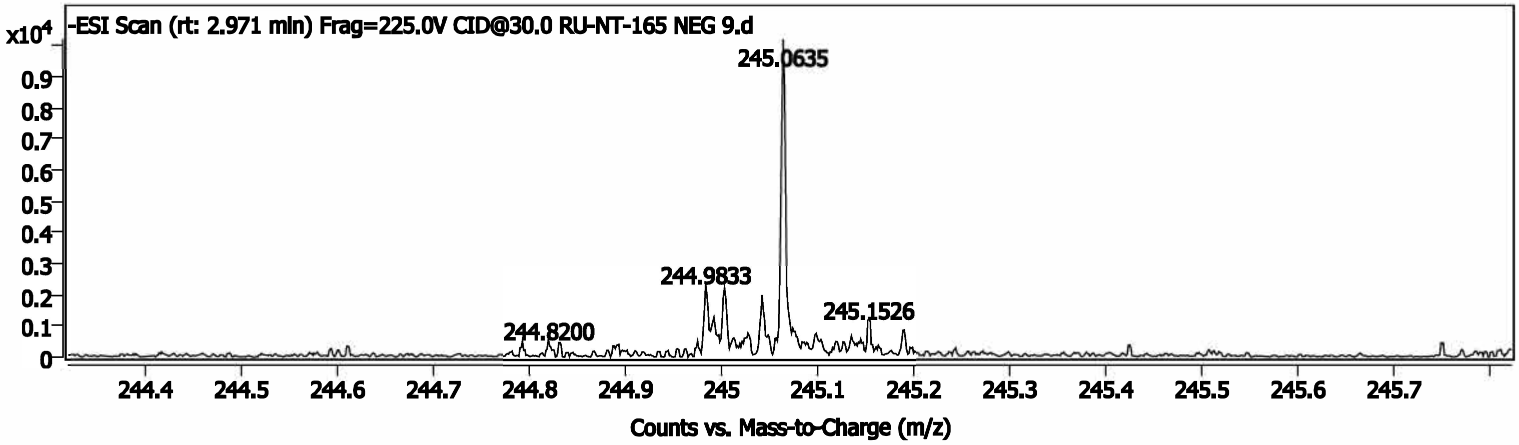

 Generated at 12:54 PM on 11/22/2024

RU-NT-192 HRMS

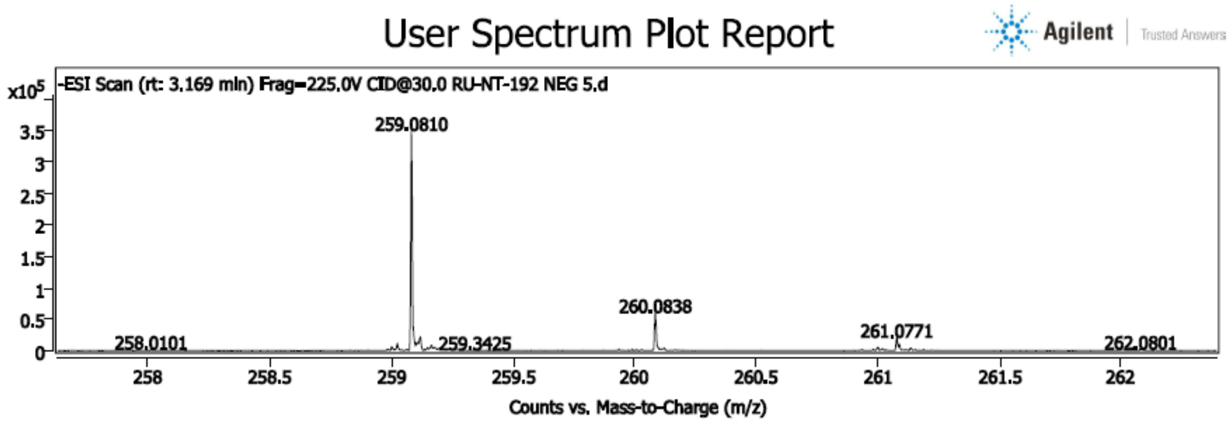

RU-NT-202 HRMS

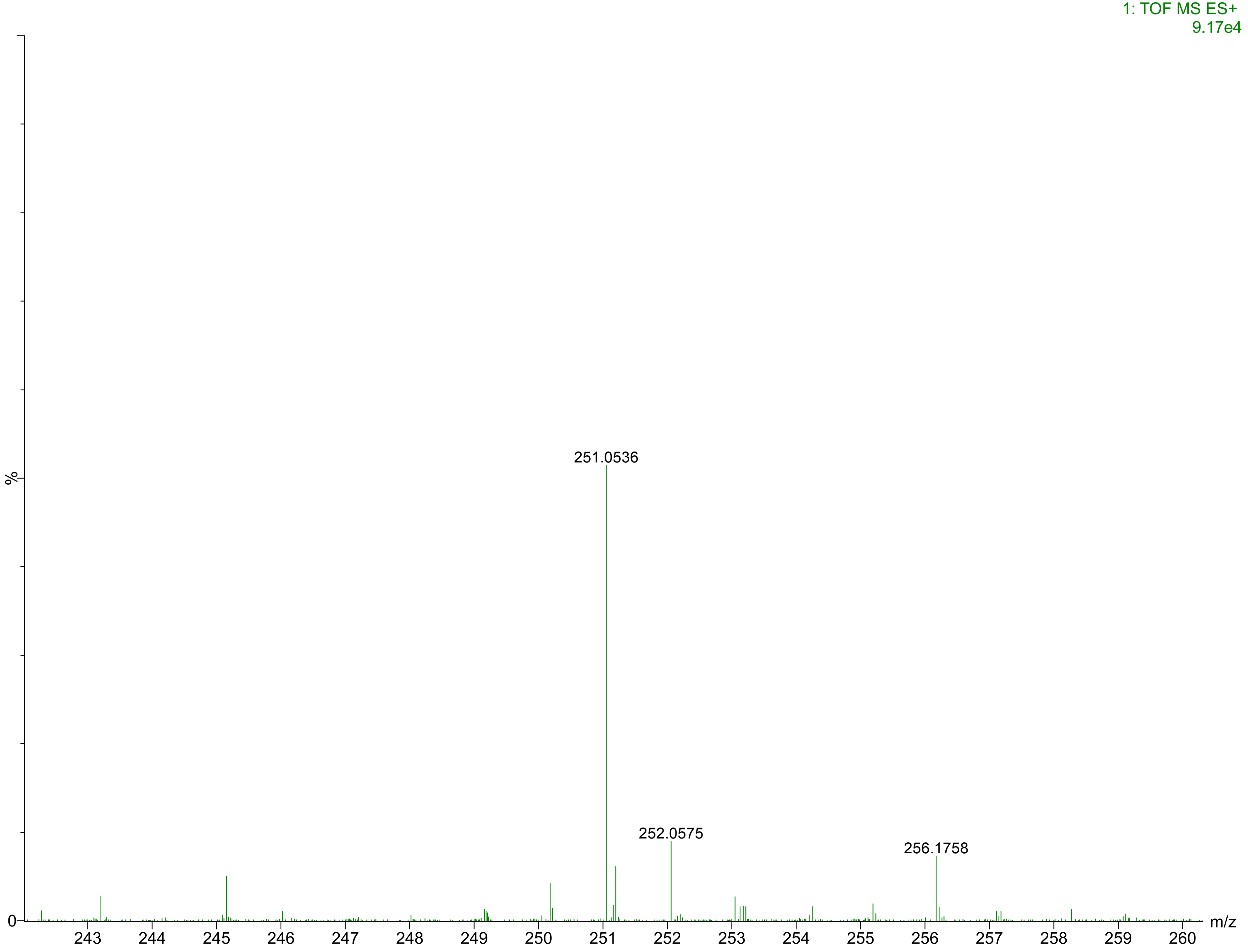

Supplement: Supporting information [file mmc1.pdf]
